# Whole-genome resequencing reveals genetic divergence, local adaptation, and conservation priorities in three helmet catfishes (complex *Cranoglanis bouderius*)

**DOI:** 10.1186/s12864-026-12843-3

**Published:** 2026-04-14

**Authors:** Shaolin Xie, Yun Hu, Jiantao Hu, Dongjie Wang, Aiguo Zhou, Yusen Li, Bo Huang, Vanthu Giap, Tuan Anh Trieu, Chenhao Zou, Chenhong Li

**Affiliations:** 1 College of Marine Sciences, South China Agricultural University, Guangzhou, Guangdong 510642 China; 2 Shanghai Universities Key Laboratory of Marine Animal Taxonomy and Evolution, Shanghai Ocean University, Shanghai, 201306 China; 3 Engineering Research Center of Environmental DNA and Ecological Water Health Assessment, Shanghai Ocean University, Shanghai, 201306 China; 4 Department of Computer Science, Lakehead University, Thunder Bay, ON P7B 5E1 Canada; 5 Guangxi Zhuang Autonomous Region, Engineering Research Center of Hongshui River Rare Fish Conservation, Guangxi Academy of Fishery Sciences, Nanning, 530021 China; 6 Faculty of Natural Sciences, Hung Vuong University, Nong Trang, Viet Tri, Phu Tho, Vietnam

**Keywords:** *Cranoglanis bouderius*, Helmet catfish, Whole-genome resequencing, Genetic divergence, Adaptive evolution, Conservation genomics

## Abstract

This study employed whole-genome resequencing to investigate the genetic diversity, population structure, and adaptive evolution of three geographically isolated populations of helmet catfish (*Cranoglanis bouderius*). These populations are distributed across the Pearl River Basin (CB) in mainland China, Hainan Island (CM), and the Red River Basin in Vietnam (CH). Despite minimal observable morphological divergence, our analysis revealed significant genetic differentiation among the populations. The CM and CH populations exhibited closer genetic affinity, likely resulting from historical biogeographic connections through the Red River Fault Zone. In contrast, the CB population was genetically more distinct. Genome-wide single nucleotide polymorphism (SNP) analysis indicated that the CB population possesses the lowest genetic diversity, while the CH population possesses the highest. This pattern is inconsistent with previous findings based on mitochondrial DNA (mtDNA). Demographic history reconstruction using the Pairwise Sequentially Markovian Coalescent (PSMC) model suggested that all three populations experienced declines during the Pleistocene glaciations. However, the CB population exhibited a more pronounced post-glacial recovery, potentially due to its inland distribution. Estimates of recent effective population size (Nₑ) from the software GONE, spanning 50 to 750 generations, revealed a contrasting trend. The CH population currently maintains a larger Nₑ than the CB population. This combination of a smaller recent Nₑ and reduced genetic diversity in the CB population underscores the urgent need for targeted conservation strategies to ensure its long-term persistence. Functional enrichment analysis (KEGG) further identified distinct adaptive signatures among the populations. Adaptive evolution in viral and immune-related pathways was detected in the CB and CH populations, likely shaped by elevated pathogen pressure in their habitats. Conversely, the CM population showed adaptive enrichment in pathways associated with metabolism and nervous system development. This finding is consistent with its more isolated and resource-constrained environment. These results highlight the profound role of long-term geographic isolation and environmental selection in driving genetic and functional divergence. They also provide critical insights for the informed conservation and management of *C. bouderius* populations.

**Supplementary Information:**

The online version contains supplementary material available at 10.1186/s12864-026-12843-3.

## Introduction

The helmet catfish (*Cranoglanis* spp.), also known as the bonefish or horse-faced bonefish, is a teleost fish belonging to the family Cranoglanididae (order Siluriformes). Endemic to East Asia, its distribution spans southern China and northern Vietnam, encompassing the Pearl River system (Guangdong, Guangxi, Guizhou), the watersheds of Hainan Island, the Yuanjiang River system (Yunnan), and the Red River system in northern Vietnam, which represents the lower reaches of the Yuanjiang River [1–3]. The family Cranoglanididae comprises a single described genus, *Cranoglanis*, which includes three geographically isolated populations or putative species: *C. bouderius*, *C. multiradiatus*, and *C. henrici*. However, their taxonomic status has long been contentious.

Early taxonomic work by Ng et al. (2000), based on traditional morphometric comparisons, recognized all three as valid species [3]. In contrast, a subsequent morphometric study by Liu et al. (2005) challenged this classification. By analyzing a larger sample size and an expanded set of anatomical landmarks, they argued for synonymizing all three into a single species, *C. bouderius* [4]. Genetic evidence from Cheng et al. (2009), using amplified fragment length polymorphism (AFLP) markers, further corroborated this synonymy, specifically supporting the unification of *C. bouderius* and *C. multiradiatus* [5]. Consistent with these findings, our previous work utilizing multiple mitochondrial DNA (mtDNA) markers (COI, Cytb, and D-loop) revealed minimal genetic divergence among the three populations, providing additional support for their recognition as a single species [6, 7]. Despite this cumulative evidence from morphology and genetics, the taxonomic status of *Cranoglanis* remains unresolved; it is still unclear whether it constitutes one species or three distinct species.

The helmet catfish is prized for its flavor and high lipid content, historically ranking among the most important edible catfish species in southern China. However, due to overfishing and habitat degradation, wild populations have experienced a sharp decline, leading to its classification as a vulnerable species in the *Chinese Red Data Book of Endangered Animals* (1998) [8]. By 2016, both *C. bouderius* and *C. multiradiatus* were reclassified as endangered in the *Red List of China's Vertebrates* [9].

Genetic diversity is a cornerstone of effective conservation strategies, yet most prior studies on helmet catfish have relied solely on mtDNA markers (e.g., COI, Cytb, D-loop). These markers have consistently revealed exceptionally low genetic diversity across all three populations, with *C. multiradiatus* exhibiting the most severe depletion of genetic variation [7, 10, 11]. Such findings underscore the urgent need for comprehensive genomic assessments to inform effective conservation actions, as current data indicate that wild helmet catfish populations are genetically vulnerable and face a high risk of further decline. In contrast, genome-wide genetic variations generated via next-generation sequencing (NGS) offer robust data for estimating population demographic parameters, detecting cryptic population structure, and resolving fine-scale genetic differentiation in fish species [12, 13].

In the present study, we developed a novel resource of genome-wide single nucleotide polymorphisms (SNPs) for helmet catfish (*Cranoglanis* spp.) via NGS, sequencing 70 individuals representing its three major populations across its distribution range in southern East Asia. This study had two interrelated objectives that prioritized foundational insights before applied implications. First, we aimed to analyze population structure and genetic differentiation among these groups to clarify long-standing taxonomic uncertainties in the *Cranoglanis* complex, thereby addressing a key knowledge gap that has hindered targeted conservation planning. Second, building on this foundational understanding, we sought to estimate the genetic diversity and dynamics of effective population sizes (Nₑ) of the three populations, thereby laying a rigorous scientific foundation for their long-term conservation and sustainable management.

## Materials and methods

### Sample collection and tissue preparation

**Fig. 1:**
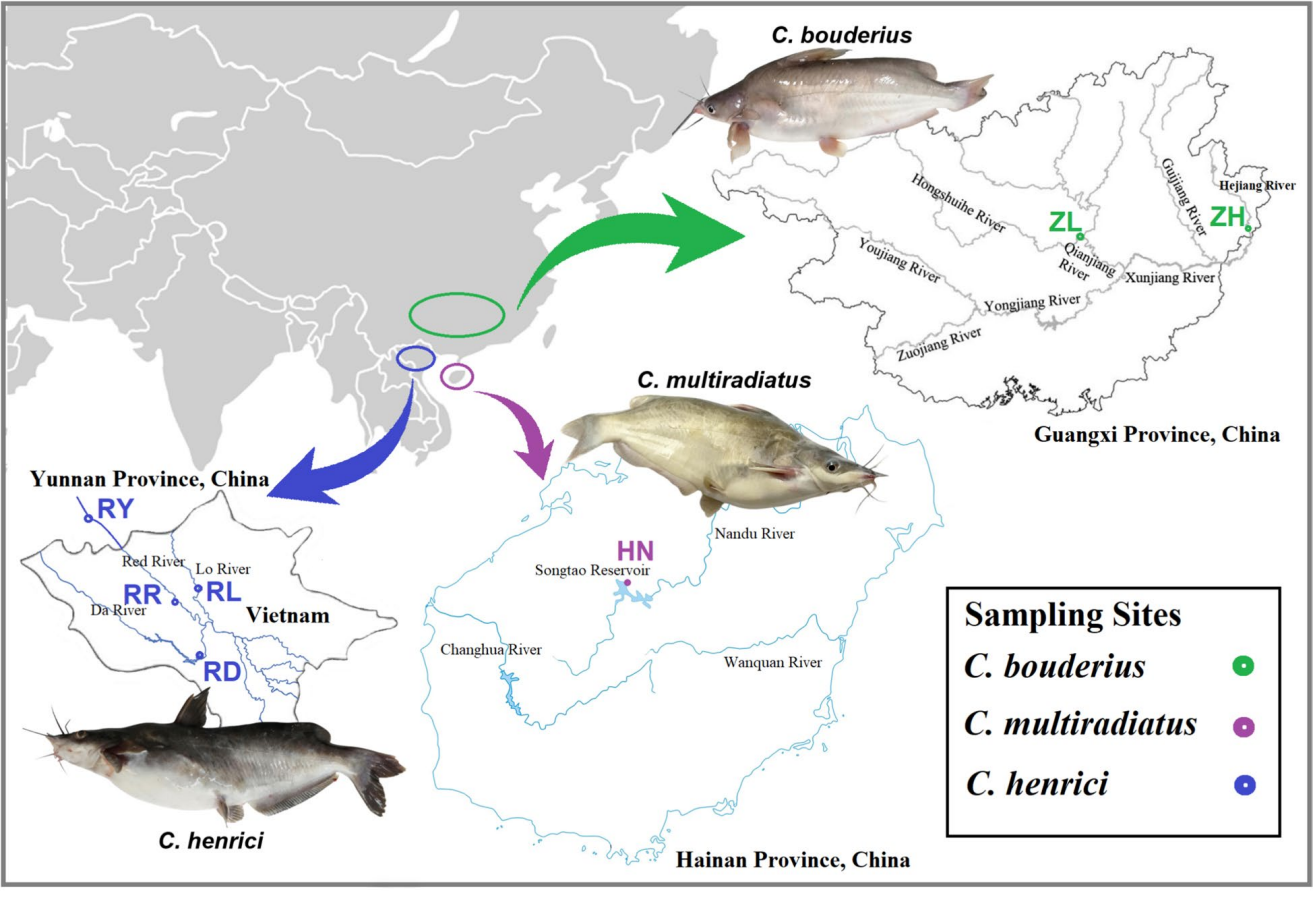
Sampling sites of *Cranoglanis* population. The green circles represent the “*C. bouderius* group”, the purple circles represent the “*C. multiradiatus* group”, and the blue circles represent the “*C. henrici* group”

A total of 70 helmet catfish were sampled, representing three geographically distinct populations: *C. bouderius* (Pearl River system), *C. henrici* (Red River system), and *C. multiradiatus* (drainages of Hainan Island). All fish samples were acquired via communication and coordination with local fishermen. The detailed sampling locations are shown in Fig. 1 and Table 1. All individuals were identified by the morphological charascter [14], and following anesthesia by immersion in MS222 (tricaine methane sulfonate), muscle tissue samples were obtained from each specimen for subsequent genetic analysis.

**Table 1:** Collection sites and sampling information of *Cranoglanis* populations

Species	code	Sample ID	Water body	Collection site	No.of samples	Date
*C. bouderius* (CB)	ZL	ZL (1–10)	Junction of Liujiang River and Hongshuihe River	Shilong Town, Guiping City, Guangxi Province, China	10	2020.07–2020.08
	ZH	ZH (1–7)	Hejiang River	Babu District, Hezhou City, Guangxi Province, China	7	2020.07–2020.08
*C. multiradiatus* (CM)	H	H (1–20)	Songtao Reservoir	Malingpai Village, Danzhou City, Hainan Province, China	20	2020.05–2020.06
*C. henrici* (CH)	RY	RY (1–3)	Yuanjiang River	Jinping County, Honghe Prefecture, Yunnan Province	3	2020.10
	RR	RR (1–11)	Red River	Tran Yen District, Yen Bai Province, Vietnam	11	2022.04–2022.05
	RL	RL (1–10)	Lo river	Tuyen Quang City, Tuyen Quang Province, Vietnam	10	2022.04–2022.05
	RD	RD (1–9)	Da river	Da Bac District, Hoa Binh Province, Vietnam	9	2022.04–2022.05

### Genome DNA extraction

Genomic DNA was extracted from each sample using the following protocol. Approximately 50–100 mg of muscle was minced with sterile scissors and transferred to a preheated (56 °C) 2.0 mL microcentrifuge tube containing 1 mL lysis buffer supplemented with 100 μL proteinase K (20 mg/mL) and 100 μL SDS (20%). The samples were incubated at 56 °C for 1–2 h with intermittent vortexing to promote digestion. Following incubation, samples were centrifuged at 12,000 rpm for 5 min at room temperature. The supernatant was carefully transferred to a fresh tube and subjected to phenol–chloroform extraction using an equal volume of phenol/chloroform/isopentanol (25:24:1), followed by centrifugation at 10,000 rpm for 10 min. The supernatant was then transferred into a new tube, and DNA was precipitated by adding 2/3 volume of isopropanol and 100 μL sodium acetate trihydrate (3 M, pH 5.2). Next, the mixture was gently inverted to mix and incubated at −20 °C for ≥ 2 h to complete precipitation. Precipitated DNA was collected by centrifugation at 10,000 rpm for 15 min at room temperature. The resulting pellet was washed with 1 mL of 75% ethanol and centrifuged at 10,000 rpm for 3 min. After air-drying for 5–10 min, the purified DNA was re-suspended in TE buffer (30–100 μL) and stored at −20 °C for subsequent library preparation.

### Library construction and sequencing

Genomic DNA (1 μg) was fragmented by using a Covaris system. The fragmented DNA was size-selected to an average range of 200–400 bp using magnetic bead purification. The size-selected fragments then underwent end repair and 3’ adenylation, followed by adaptor ligation. The ligated products were PCR-amplified and further purified with magnetic beads. Finally, the double-stranded PCR products were subsequently heat-denatured and circularized via splint oligo sequence. The single strand circle DNA (ssCirDNA) were formatted as the final library and qualified by QC. The final libraries were amplified with phi29 polymerase to make DNA nanoballs (DNBs), each containing > 300 molecular copies. The DNBs were load onto patterned nanoarray and sequenced by using MGISEQ-2000 sequencer, generating 100–150 bp paired-end reads through combinatorial Probe-Anchor Synthesis (cPAS) technology.

### Quality control of raw sequencing reads

Following sequencing, raw reads underwent quality control processing to remove adaptor sequences, contaminants and low-quality reads. This step was completed using SOAPnuke software v2.1.8 (BGI) with the following parameters: -n 0.01 -l20 -q 0.3 –adaMR 0.25 –ada_trim –polyX 50 –minReadLen 150 [15].

### SNP calling and population genetic metrics

Cleaned sequencing data from each sample (*n* = 70) were aligned to the reference genome of helmet catfish (accession No.: GCA_026119655.1) using bwa v0.7.16a-r1181 with default parameters [16], followed by conversion of alignment files into binary BAM format using SAMtools v1.21 [17]. PCR duplicates were removed using the Picard v3.3.0 (http://broadinstitute.github.io/picard). GATK v4.05.1 was used to genotype the SNP loci [18]. Raw SNP sites were filtered with VCFtools v0.1.16 (Danecek et al., 2011) to exclude loci with more than two alleles (–max-alleles 2), those with a Minor Allele Frequency less than 0.05 (–maf 0.05) and those genotyped with missing individuals are excluded (–max-missing 1.0). As linked sites are redundant for population structure and gene flow inference, the filtered dataset was subjected to linkage disequilibrium (LD) pruning with PLINK v1.9 with the parameter “–indep-pairwise 50 5 0.5” to extract an LD-pruned SNP set before downstream analysis.

Population genetic metrics, including pairwise population differentiation (*F*_*ST*_) and nucleotide diversity (π) of both variant and all sites were calculated using the populations v1.44 in Stacks program [19]. The degree of differentiation between two populations were assessed according to the *F*_*ST*_ values [20].

### Population clustering and genetic structure

The aligned DNA sequences (see "SNP calling and population genetic metrics" section) were concatenated for phylogenetic tree inference using the maximum likelihood method (ML) to reveal the relationship of the 70 individuals of *Cranoglanis* along with two *Pangasianodon hypophthalmus* specimens (SRR25572294 and SRR18779818) as outgroups. The ML tree was reconstructed using RAxML v8.0.0 under the GTRGAMMA model with 1000 bootstraps replicates [21] and visualized using Figtree v1.4.2 (http://tree.bio.ed.ac.uk/software/Figuretree/).

The population structure of the 70 individuals of *Cranoglanis* was examined using the ADMIXTURE software v1.3.0 with default parameters. The number of clusters (K) being set from 1 to 7 according to the numbers of geographical sampling sites [22]. The optimal number of genetic clusters was defined by the K with the lowest CV error [23].

Additional Principal Component Analysis (PCA) was performed using PLINK v1.9, with the maximum number of principal components (PCs) set to 7. The results for the first two principal components (PC1 and PC2) were visualized in a scatter plot using the ggplot2 package in R v4.4.1.

### History of population divergence analysis

The demographic history including demographic fluctuations and gene flow events among the three populations of *Cranoglanis* were performed by using the joint site frequency spectrum (SFS)-based approach implemented in fastsimcoal2 [24]. Python scripts easySFS.py (https://github.com/isaacovercast/easySFS) were used to calculate the joint site frequency spectrum. Cluster analysis revealed three distinct genetic groups of *Cranoglanis* corresponding to these three populations: CB, CM and CH. Based on these findings, the evolutionary history of these three groups was evaluated using the following models: models 1 to 3 served as initial models, each hypothesizing that either CB, CM, or CH diverged first from the other two groups under a strict isolation (SI) scenario. The best-performing model among these three was then extended by incorporating different migration scenarios: Isolation-with-Migration (IM, model 4), Ancient Migration (AM, model 5), and Secondary Contact (SC, model 6) between the different groups. These models were evaluated using fastsimcoal2 v2.7, following analytical procedures established in our previous study [20].

### Linkage disequilibrium and effective population size

To evaluate Linkage Disequilibrium (LD) decay, the Pearson’s squared correlation coefficient (r2) between each pair of markers was calculated using PopLDdecay [25]. Pairwise Sequential Markovian Coalescent (PSMC) analysis on five representative individuals per each population were performed to infer the history of the effective population size (N_e_) changes in the ancestors of the three *Cranoglanis* populations over the last 5 million years (Ma) [26]. The PSMC v0.6.5-r67 was used and parameters were set as follows: − N25, − t15, − r5 and − p '4 + 25*2 + 4 + 6'. Given PSMC is limited in its ability to detect recent effective population sizes, recent effective population size (N_e_) changes on three *Cranoglanis* populations were estimated using the software package GONe v1.03 with default parameters.

### Genome-wide selective sweeping analysis

Genome-wide scans were conducted for signatures of positive selection by analyzing patterns of genetic variation across populations. Nucleotide diversity was quantified using pairwise θ_π_ estimates, while genetic differentiation was assessed through pairwise *F*_*ST*_ calculations. Both metrics were computed using a sliding window approach. We optimized the window parameter by first quantifying the LD decay pattern of the study populations: the window size was set to ~ 20 kb (the physical distance at which pairwise linkage disequilibrium, measured as r^2^, decayed to a threshold of 0.1), which ensures the inclusion of genetically linked loci while avoiding redundant signals from weakly linked regions beyond the LD decay distance. The sliding window was advanced in 20 kb increments, consistent with the original step size to maintain resolution for capturing spatial genetic variation. Genomic regions under selection were identified by intersecting the top 5% of *F*_*ST*_ values with the top 5% and bottom 5% of θ_π_ ratio values, yielding two distinct sets of candidate genomic regions; genes residing within these intervals constitute the respective selective targets of each population. To consider the influence of population structure on adaptive loci detection, we supplemented the above analysis with pcadapt to refine the candidate SNPs. Specifically, we used pcadapt v4.4.0 to detect and filter out SNPs potentially confounded by population structure, thereby minimizing false positives arising from strong genetic differentiation. Only the SNPs that passed pcadapt filtering were retained as high-confidence candidate adaptive loci, and the corresponding genes within their intervals were finalized as selective targets for subsequent functional annotation and validation. Genes located within these candidate regions were extracted using BEDTools v2.26.0 and subsequently annotated against the NR (NCBI, accessed 2025–01–28), UniProtKB/Swiss-Prot 2025_01, KOG, PFAM 37.0, Gene Ontology (GO, accessed 2025–01–28) and KEGG (Release 115.0) databases using BLAST 2.17.0. Functional enrichment analysis of Gene Ontology (GO) and KEGG pathways were conducted using the OmicShare platform, a freely accessible online tool for omics data analysis (http://www.omicshare.com/tools/, accessed on 2025–02–20) [27].

## Results

### Whole genome resequencing of *Cranoglanis* populations

A total of 70 helmet catfish individuals were collected for whole-genome resequencing: 17 individuals from the Pearl River system, 20 individuals from Hainan’s Songtao Reservoir, and 33 individuals from the Red River watershed (Fig. 1 and Table 1). Sequencing achieved an average depth of 20 × coverage across genomes, generating approximately 77.8 million clean reads per individual after quality filtering (23.34 Gb clean data per sample; Supplementary Tab. S1). A total of 13,680,252 raw SNPs were initially called from resequencing genome data. Following rigorous quality control (QC) filtering, 2,500,402 high-quality SNPs were retained. Subsequent LD pruning to mitigate redundancy from linked loci yielded a final set of 443,421 LD-pruned SNPs, which served as the core dataset for all subsequent downstream genetic analyses.

### Genetic population structure of the *Cranoglanis* individuals

**Fig. 2:**
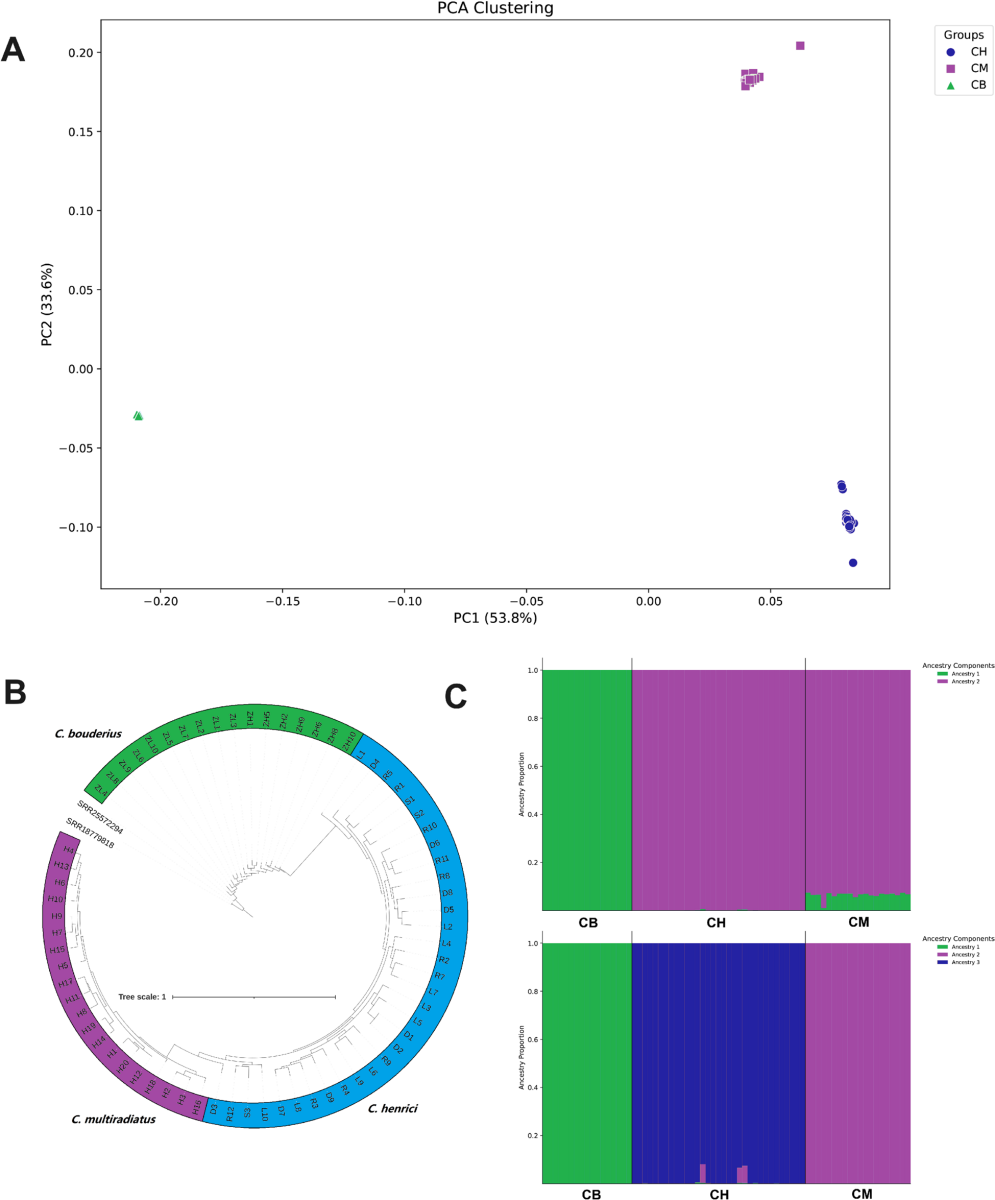
**A** Principal Component Analysis (PCA) scatterplot showing genetic clustering of 70 individuals into three distinct groups: green, *C. bouderius* (CB), blue, *C. Henrici* (CH), and purple, *C. multiradiatus* (CM); **B** Maximum likelihood tree constructed using RAxML (GTRGAMMAR mode with 1,000 bootstrap replicates) based on captured gene sequences. **C** The results of STRUCTURE analysis of three *Cranoglanis* populations

We employed multiple analytical approaches to investigate the genetic structure of helmet catfish populations. PCA identified three distinct genetic clusters corresponding to three species: (1) *C. bouderius* (CB), (2) *C. henrici* (CH), and (3) *C. multiradiatus* (CM) (Fig. 2A). Notably, CH and CM populations showed closer genetic affinity to each other than to CB. Consistent with these findings, ML phylogenetic reconstruction demonstrated that CB formed the most divergent clade (green, Fig. 2B), while CM (violet) and CH (blue) clustered together in a separate clade. This pattern was further supported by STRUCTURE analysis, which identified K = 3 as the optimal clustering solution (Fig. 2C). At K = 2, CH and CM populations merged into a single genetic group, confirming their close evolutionary relationship. The concordance among PCA, ML tree, and STRUCTURE analyses provides robust evidence for the observed population structure.

### Historical population dynamics of *Cranoglanis*

The demographic history of *Cranoglanis* including population fluctuations and gene flow events among the three studied populations was inferred using a joint site frequency spectrum (SFS)-based approach implemented in *fastsimcoal2*. Two likelihood measures, MaxObsLhood (maximum observed likelihood) and MaxEstLhood (maximum estimated likelihood), were generated. Six demographic models were tested, encompassing initial strict isolation (SI) scenarios and extended migration-inclusive frameworks, with model performance evaluated using maximum likelihood metrics (MaxObsLhood, MaxEstLhood, Delta-Likelihood) and the Akaike Information Criterion (AIC; Table 2).

**Table 2:** Model comparison of *Cranoglanis* population divergence history inferred from site frequency spectrum (SFS)

Models	MaxEstLhood	MaxObsLhood	Delta-Likehood	AIC
Model 1	−10,125,601.11	−8,686,412.929	−1,439,188.183	46,658,779.91
Model 2	−10,181,928.25	−8,686,412.929	−1,495,515.325	46,918,335.38
Model 3	−10,130,044.19	−8,686,412.929	−1,443,631.262	46,679,253.63
Model 4	−9,715,279.664	−8,686,412.929	−1,028,866.735	44,768,030.69
Model 5	−9,641,295.976	−8,686,412.929	−954,883.047	44,427,115.86
Model 6	−9,426,923.77	−8,686,412.929	−740,510.841	43,439,288.73

Notably, all six models yielded an identical maximum observed likelihood (MaxObsLhood = −8,686,412.929), while substantial variation was observed in maximum estimated likelihood (MaxEstLhood) and AIC values. The initial strict isolation models (models 1–3), each hypothesizing a distinct first divergence event (CB, CM, or CH diverging first from the other two clades), exhibited relatively high AIC values (ranging from 46,658,779.91 to 46,918,335.38) and large Delta-Likelihood values (ranging from −1,439,188.183 to −1,495,515.325), indicating poor fit to the jSFS data. In contrast, the extended models incorporating migration (models 4–6: Isolation-with-Migration [IM], Ancient Migration [AM], and Secondary Contact [SC]) outperformed the SI models, with lower AIC and Delta-Likelihood values. Among all tested models, model 6 (Secondary Contact) achieved the lowest AIC (43,439,288.73) and Delta-Likelihood (−740,510.841), coupled with a MaxEstLhood of −9,426,923.77, thereby emerging as the best-supported model for reconstructing the demographic history of *Cranoglanis*.

**Fig. 3:**
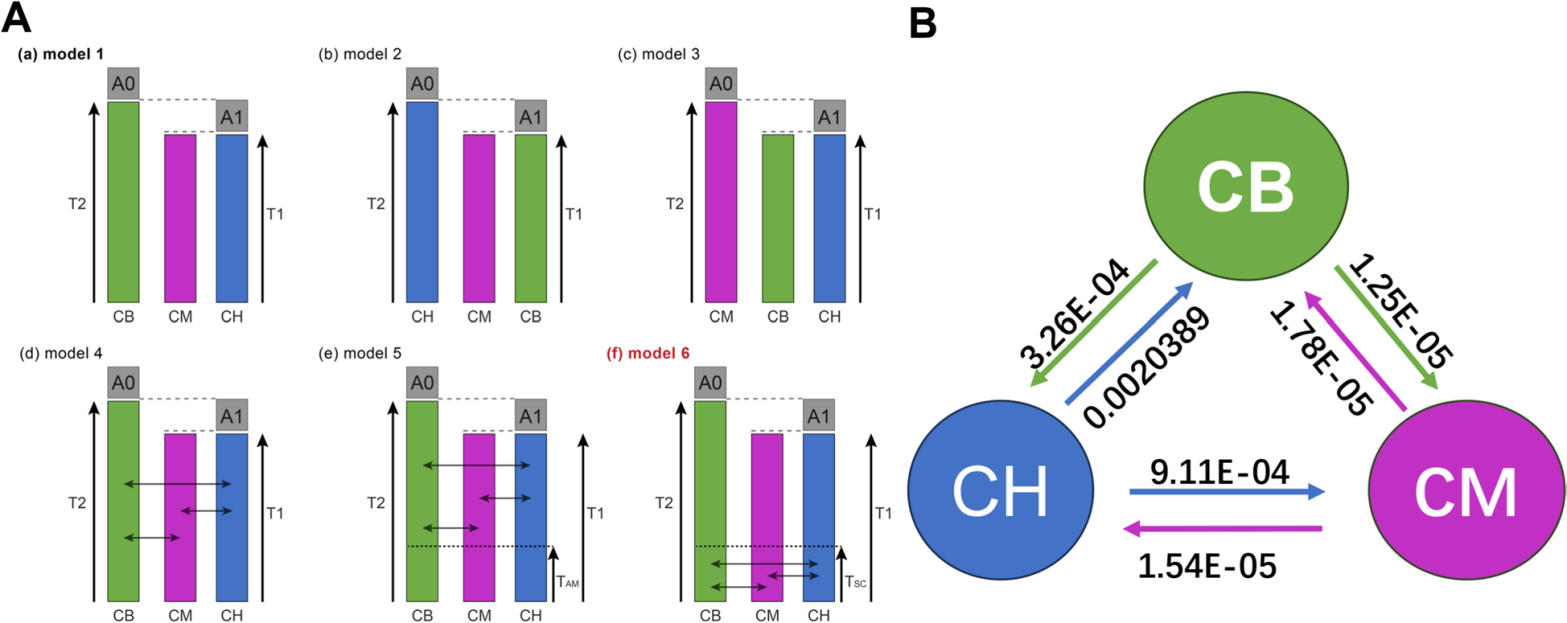
Demographic models of three *Cranoglanis* populations. **A** Divergence and migration scenarios: (a) Model 1 (strict isolation, SI): *C. bouderius* (CB) diverged first from the common ancestral population (A0), followed by the subsequent split of *C. multiradiatus* (CM) and *C. henrici* (CH); (b) Model 2 (strict isolation, SI): CM was the first lineage to diverge from ancestor A0, with CB and CH splitting later; (c) Model 3 (strict isolation, SI): CH diverged first from ancestor A0, followed by the divergence of CB and CM; (d) Model 4 (Isolation-with-Migration, IM): Extended from the strict isolation framework with incorporated contemporary gene flow among the three populations; (e) Model 5 (Ancient Migration, AM): Modified from initial SI models, incorporating gene flow events that occurred only during the early stage of population divergence; (f) Model 6 (Secondary Contact, SC): Hypothesizes initial strict isolation of the three lineages followed by subsequent episodes of secondary gene flow. **B** Estimated gene flow intensity between adjacent clades. The numerical values on the connecting lines represent the strength of gene flow between the corresponding populations, with the magnitude reflecting the extent of genetic exchange

Consistent with the structure of model 6 (Fig. 3A), the inferred evolutionary trajectory indicates that the common ancestral population (A0) first split into the CB lineage and a second ancestral lineage (A1), which subsequently diverged into the CH and CM clades. Statistically significant gene flow events were detected among all three clades (Fig. 3B), and the genetic exchange patterns were further refined based on bidirectional migration rate estimates and effective migration rates (Nm = migration rate [m] × effective population size [N_e_]) derived from this best-supported Secondary Contact model: The highest raw migration rate (m) was observed in the CH → CB direction (estimated parameter value = 0.002039), followed by the CH → CM direction (9.11 × 10⁻^4^), while the lowest raw migration rates were recorded for CB → CM (1.25 × 10⁻^5^) and CM → CH (1.54 × 10⁻^5^). When quantifying biologically meaningful genetic exchange using Nm (a metric that integrates migration intensity and population size to reflect the number of effective migrants per generation [28], the CH → CB pair exhibited the strongest connectivity (Nm ≈ 1.03) (Tab. S3). This value approaches the well-recognized threshold of 1 for maintaining genetic homogeneity. It was followed by CH → CM (Nm ≈ 0.46), indicating limited yet detectable gene flow. In contrast, all bidirectional Nm values for CM ↔ CB (CB → CM: ~ 0.004; CM → CB: ~ 0.007) and CM → CH (CM → CH: ~ 0.006) were substantially below 1, confirming that CM exhibits almost no functional genetic exchange with the other two clades.

### Genetic diversity and differentiation

Our analysis of population genetic diversity uncovered distinct patterns across the three studied groups (CB, CM, and CH). In terms of nucleotide diversity (π), CB populations exhibited the lowest values, with ZL and ZH subpopulations showing π = 0.0723 and π = 0.0689, respectively (Table 3). In contrast, CM and CH populations displayed comparable levels of genetic diversity, with the CH population (RR subpopulation) achieving the highest π value (0.2965) among all groups (Table 3).

**Table 3:** Nucleotide diversity (π) estimates for each *Cranoglanis* population, including both variant and all genomic positions

Species	Code	pi (variant positions)	All positions
*C. multiradiatus*(CM)	HN	0.2392	1.3001921E-04
*C. bouderius*(CB)	ZL	0.0723	1.0621788E-04
	ZH	0.0689	1.3161781E-04
*C. henrici*(CH)	RY	0.2922	1.3166221E-04
	RR	0.2965	1.2975278E-04
	RL	0.2964	3.0595367E-05
	RD	0.2928	3.2105153E-05

Complementary analysis of individual heterozygosity revealed a contrasting pattern relative to nucleotide diversity (Table 3): the CH population (*n* = 34) had a mean heterozygosity index of −0.1211 (range: −0.1949 to −0.0613); the CM population (*n* = 20) exhibited a mean index of −0.0048 with substantial inter-individual variation (range: −0.5602 to 0.1115); and the CB population (*n* = 17) showed the highest mean heterozygosity index (0.7072) with a relatively narrow range (0.6401 to 0.7305) (Tab. S4 and Supplementary material individual heterozygosity).

Population differentiation assessed via pairwise *F*_*ST*_ comparisons further delineated the genetic relationships among these groups (Table 4). Substantial genetic divergence was observed between CB and CH populations (*F*_*ST*_ range: 0.0817701–0.185934), which significantly exceeded the moderate differentiation detected between CM and CH (*F*_*ST*_ range: 0.0420637–0.0527671). Collectively, these *F*_*ST*_ patterns indicate stronger genetic isolation between CB and the other two populations, whereas CM and CH maintain a relatively close genetic relationship.

**Table 4:** Population pairwise genetic differentiation (*F*_*ST*_) estimates among *Cranoglanis* groups

	HN	ZL	ZH	RY	RR	RL	RD
HN		0.109418	0.0914943	0.0420637	0.0516314	0.0505145	0.0527671
ZL	0.0		0.0138374	0.185934	0.10657	0.107186	0.122685
ZH	0.0	0.0		0.137307	0.0817701	0.0887219	0.0929072
RY	0.0	0.0	0.0		0.00563246	0.00605307	0.00584558
RR	0.0	0.0	0.0	0.0		0.00499917	0.00577269
RL	0.0	0.0	0.0	0.0	0.0		0.0067924
RD	0.0	0.0	0.0	0.0	0.0	0.0	

### Linkage disequilibrium and effective population size

**Fig. 4:**
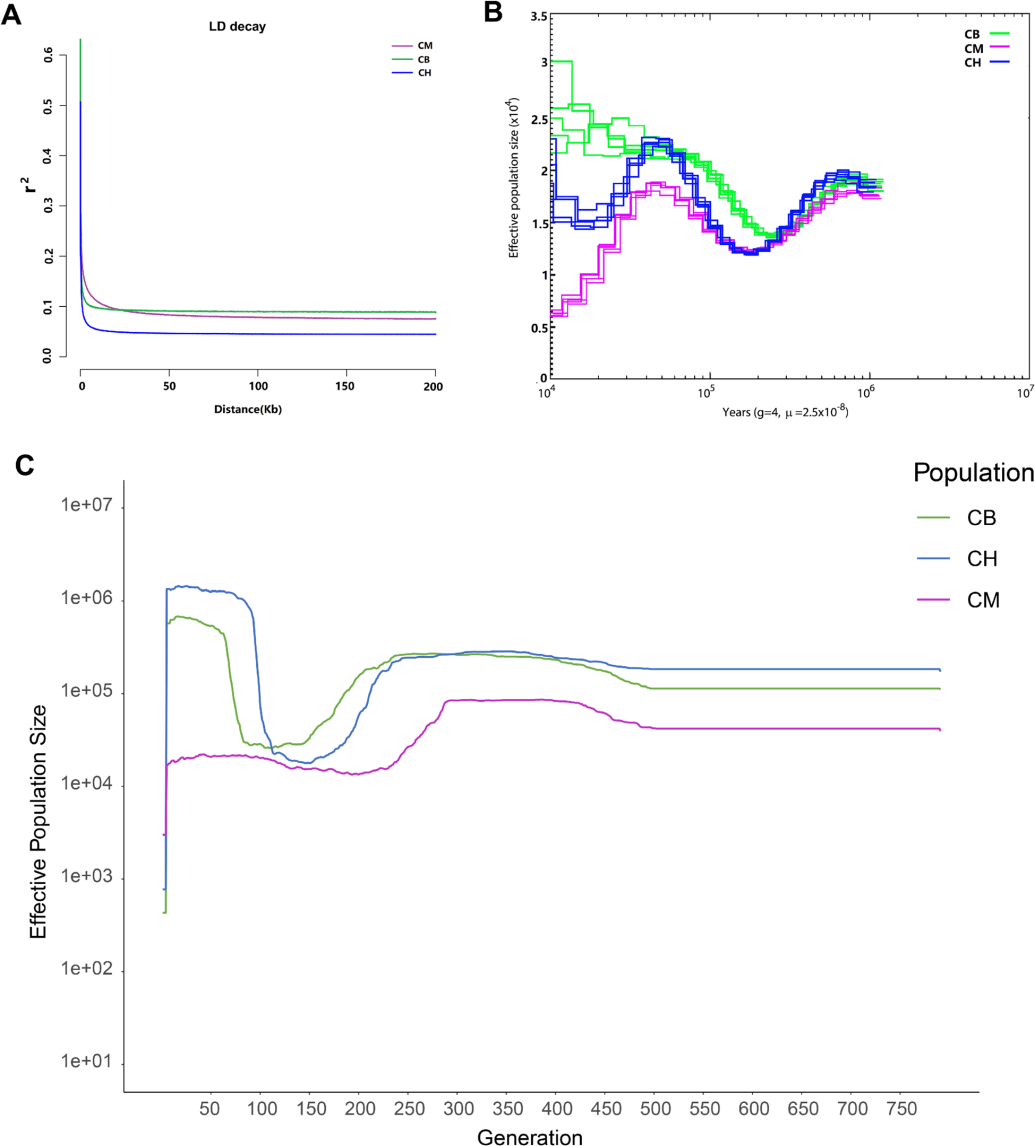
**A** Average linkage disequilibrium decay by physical distance for three *Cranoglanis* population. **B** Historical changes in effective population size reconstructed through pairwise sequentially markovian coalescent (PSMC) analysis of individual whole genomes, with five randomly selected individuals analyzed per population. **C** Recent changes in effective population size (N_e_) across 50–750 generations for the three populations, inferred using the GONE software (which targets short-term N_e_ dynamics via genome-wide linkage disequilibrium patterns)

Linkage Disequilibrium (LD) decay was estimated for the three *Cranoglanis* populations base on the pairwise correlations of markers with 0–200 kb intervals. Figure 4 illustrates the decay of r^2^ as a function of distance for three *Cranoglanis* populations. The results showed that the decay was slower in CM (< 15.5 Kb) than other two populations CB (< 6.6 kb) and CH (< 1.1 kb) when the LD decay drop below the threshold for r^2^ = 0.1 (Fig. 4A). In addition, CB exhibited a higher baseline LD (r^2^ = 0.087) than CM (r^2^ = 0.075).

PSMC analysis revealed a decline in effective population size (Nₑ) for all three populations beginning 1 million years ago (Mya). The lowest Nₑ values occurred at different time points: CB reached a trough (Nₑ ∼1.35 × 10^4^) at 0.2–0.3 Mya, while CH and CM reached their minima (Nₑ ∼1.15 × 10^4^) slightly later, at 0.1–0.2 Mya (Fig. 4B). Subsequently, Nₑ began to recover gradually in all populations. The N_e_ of CH was relatively stable and had been on an upward trend. While CH maintained relatively stable growth, CH and CM both peaked (∼2.4 × 10^4^∼1.9 × 10^4^, respectively) at 35–50 thousand years ago (ka), followed by another decline (Fig. 4B). Notably, by 10 ka, CM had the lowest Nₑ (∼6,000) among the three populations.

Recent effective population size (N_e_) of three *Cranoglanis* populations (CB, CH, CM) was estimated using GONE, covering 50–750 recent generations (log₁₀-scaled y-axis for N_e_). A clear hierarchical and stable pattern emerged: CH maintained the largest N_e_ (1 × 10⁶–1 × 10⁷), followed by CB with intermediate N_e_ (1 × 10^5^–1 × 10⁶), and CM with the smallest N_e_ (1 × 10^4^–1 × 10^5^) (Fig. 4C). No significant fluctuations, expansions, or contractions were detected for any population over the observed timeframe across all three groups.

### Analysis of selective sweeping

To safeguard the reliability of candidate selected genes and mitigate false positives stemming from strong genetic differentiation driven by population structure, we first subjected SNPs to stringent filtering via pcadapt before initiating the identification of selection signatures (Fig. S1A). Following this filtering step, we generated an *F*_*ST*_ Manhattan plot to further characterize the genome-wide distribution of these putatively adaptive loci, leveraging the pairwise *F*_*ST*_ values of the filtered SNPs (Fig. S1B). Complementing these analyses, we employed SweeD v 3.2.1 to generate complementary Manhattan plots of Alpha values for each pairwise population comparison (CB vs. CH, CB vs. CM, and CH vs. CM; Fig. S2A–C). These plots revealed a non-uniform distribution of Alpha values across the 38 chromosomes, with prominent, discrete peaks of elevated Alpha values in each comparison, pinpointing distinct genomic regions under strong positive selection. Notably, these selection peaks exhibited both overlapping and unique patterns across the three population pairs (Fig. S2). For the *F*_*ST*_-based analyses, Z-transformed Fₛₜ values displayed distinct chromosomal fluctuations in each pairwise comparison, with alternating blocks representing individual chromosomes (e.g., CM047530, CM047531). In the CB vs. CH comparison, Z-transformed *F*_*ST*_ values range approximately from −1.5 to 1.5, revealing scattered genomic regions with elevated differentiation that likely harbor loci under selection (Fig. S3A). Similarly, the CB vs. CM comparison shows comparable fluctuation ranges, though the specific positions of high and low *F*_*ST*_ peaks differ, indicating population-specific selection signals (Fig. S3B). Notably, the CH vs. CM comparison displays the strongest differentiation, with Z-transformed *F*_*ST*_ values reaching up to 4, which aligns with the largest number of candidate selected genes (1,061) detected in this pair (Fig. S3C). These plots confirm that our filtered SNP set retains meaningful differentiation signals, and the uneven distribution of high Z-transformed *F*_*ST*_ values across chromosomes guided our identification of top 5% *F*_*ST*_ regions for subsequent analysis.

**Fig. 5:**
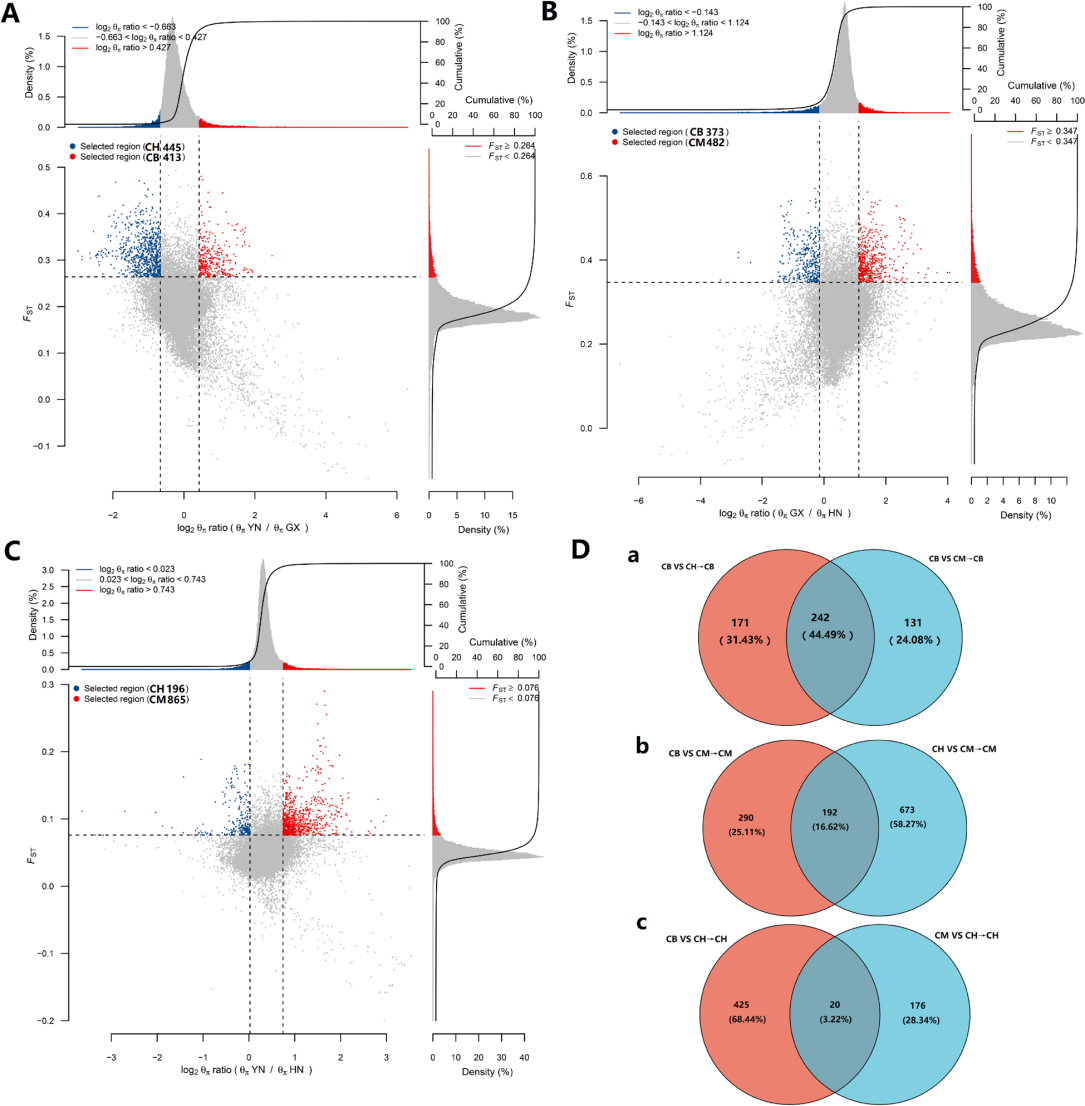
Results of genome-wide selective sweep analysis. **A** CB vs CH comparison. **B** CB vs CM comparison. **C** CM vs CH comparison. **D** Venn diagram illustrating overlap in numbers of selected genes across comparisons

Subsequently, we identified candidate selected genes by intersecting the top 5% of *F*_*ST*_ values with the top 5% and bottom 5% of θ_π_ ratio values (key metrics for defining selection signatures): 858 selected genes were detected in the CB vs. CH comparison (413 genes in CB, 445 in CH; Fig. 5A), 855 in CB vs. CM (373 in CB, 482 in CM; Fig. 5B), and 1061 in CM vs. CH (865 in CM, 196 in CH; Fig. 5C).

**Fig. 6:**
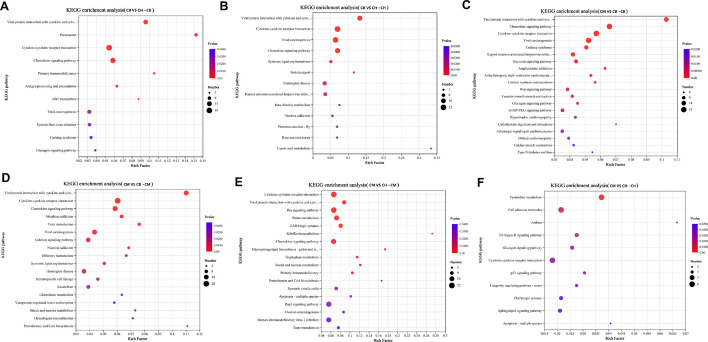
KEGG pathway enrichment analysis of differentially selected genes among three *Cranoglanis* populations. **A** and **B** show enriched pathways for selected genes in CB vs CH. **C** and **D** display enriched pathways for CB vs CM. **E** and **F** present enriched pathways for CM and CH

Comparative analysis revealed substantial overlaps between groups: CB vs. CH and CB vs. CM shared 242 identical genes (44.49% overlap) in CB (Fig. 5D a), while CB vs. CM and CM vs. CH shared 192 identical genes (16.62% overlap) in CM (Fig. 5D b). In contrast, CB vs. CH and CM vs. CH had just 20 identical genes (3.22%) in CH (Fig. 5D c). No genes were common to all three comparisons (Fig. S4). GO and KEGG enrichment analyses of the selected genes revealed distinct functional profiles (Fig. S5 and Fig. 6). For CB-specific comparisons (CB_VS_CH_CB and CM_VS_CB_CB), we observe significant enrichment in viral interaction pathways (e.g., viral protein interaction with cytokines, viral carcinogenesis) and immune response pathways (e.g., cytokine-cytokine receptor interaction, chemokine signaling). The CM-specific comparisons (CM_VS_CH_CM and CM_VS_CB_CM) showed broader functional enrichment, including metabolic pathways (e.g., purine metabolism, starch and sucrose metabolism) and nervous system-related pathways (e.g., GABAergic synapse, cholinergic synapse). In contrast, CH-specific comparisons (CB_VS_CH_CH and CM_VS_CH_CH) demonstrated weaker pathways enrichment factors, suggesting less pronounced selective in these comparisons.

## Discussions

### Validity of the species of the genus *Cranoglanis*

The taxonomic status of the genus *Cranoglanis* remains controversial, with persistent debate regarding its classification as a single species or three distinct taxa. Prior morphological and molecular studies have yielded conflicting evidence. Several researchers have advocated for the recognition of three species (CB, CM, and CH) based on life-history traits and morphological characteristics [3, 29]. Specifically, Ng et al. (2000) explicitly endorsed this three-species classification by conducting detailed comparisons of key morphological traits, such as humeral process width, snout morphology, anal fin length, branched fin ray count, vertebral number, and interorbital distance [3]. In contrast, Liu et al. (2005) analyzed 66 *Cranoglanis* specimens via multivariate morphometric methods and concluded that the observed morphological differences were insufficient to delineate distinct species, thus recognizing only CB as valid [30]. Molecular evidence derived from AFLP markers and three mitochondrial DNA sequences has further corroborated the single-species hypothesis [5–7].

Our analysis of countable and quantifiable morphological traits revealed no significant differences among the three populations (Tab. S2). We further explored the population structure and evolutionary history of these three populations using multiple complementary analytical approaches, including PCA, phylogenetic analysis, STRUCTURE, and fastsimcoal2. All methods consistently delineated the three populations as distinct groups. PCA and phylogenetic analyses indicated that CM and CH form a cohesive cluster, a pattern corroborated by STRUCTURE analysis, which grouped CM and CH together at K = 2. Reconstruction of population demographic history showed that CH and CM diverged from a common ancestral lineage (A1), which itself originated from the ultimate common ancestor (A0) alongside the CB clade. This finding was consistent with subsequent gene flow analyses.

Bidirectional raw migration rates (m) and effective migration rates (Nm = m × N_e_) revealed substantial heterogeneity among the three clade pairs. The highest raw migration rate was recorded for CH → CB (0.002039), followed by CH → CM (9.11 × 10⁻^4^), while the lowest rates were observed for CB → CM (1.25 × 10⁻^5^) and CM → CH (1.54 × 10⁻^5^). Correspondingly, the effective migration rate between CH and CB was substantially higher than that between CH and CM or between CB and CM: CH → CB exhibited the strongest connectivity (Nm ≈ 1.03), approaching the well-recognized threshold of 1 for maintaining genetic homogeneity, followed by CH → CM (Nm ≈ 0.46), which indicates limited but detectable gene flow. In contrast, all bidirectional Nm values for CB ↔ CM (CB → CM: ~ 0.004; CM → CB: ~ 0.007) and CM → CH (Nm ≈ 0.006) were far below 1, while CB → CH showed a moderate Nm of ~ 0.11. Collectively, these results confirm that CH-CB gene flow is the most prominent, CH-CM gene flow is weaker, and CB-CM genetic exchange is functionally negligible.

These genetic results align well with the biogeographic history of the study region. Specifically, paleogeographic evidence shows that Hainan Island was contiguous with northern Vietnam and Guangxi during the Eocene, before drifting southeastward along the Red River Fault Zone from the Oligocene to Miocene [31, 32]. This shared paleogeographic history is mirrored in the striking floristic similarities between Hainan and Vietnam [33] as well as the close phylogenetic relationships observed in other taxa (e.g., *Channa gachua*, *Leiolepis reevesii*) [34, 35].

We further verified this biogeographic connection using demographic parameters inferred from the best-supported fastsimcoal2 Model 6 (Secondary Contact). All time estimates were converted to calendar years based on a 3-year generation time for *Cranoglanis*, which is consistent with life-history data from closely related catfish taxa and was validated through sensitivity analyses. Among these parameters, T2 marks the split of the common ancestral population (A0) into the CB lineage and a second ancestral lineage (A1, the common ancestor of CH and CM), estimated at 91,104 generations (~ 273,312 years ago, ya); T1 represents the subsequent divergence of A1 into CH and CM, calculated as 36,146 generations (~ 108,438 ya); and TSC denotes the onset of secondary contact among all three clades, inferred to be 1,129 generations (~ 3,387 ya) (Supplementary Excel FSCmodels). The temporal sequence of population divergence aligns closely with the region’s paleogeographic evolution: T2 (~ 273 ka), marking the split of ancestral population A0 into CB and A1 (common ancestor of CH and CM), falls within the MIS 8–7 transition—a period of global sea-level rise that submerged land bridges, isolating Hainan’s aquatic systems from the Pearl River (CB) and Red River (CM) basins [36]. T1 (~ 108 ka), corresponding to the divergence of A1 into CH and CM, coincides with MIS 5a, a warm interglacial phase with heightened monsoon intensity and further sea-level rise that exacerbated river system fragmentation, isolating the Red River (CM) from Hainan’s inland rivers (CH) [37]. TSC (~ 3.4 ka), the onset of secondary contact among the three clades, postdates the Holocene Thermal Maximum and overlaps with the early Bronze Age in southern China, when reduced monsoon intensity stabilized river flows and reestablished hydrological connections between basins [38]. All key population divergence and reconnection times are consistent with concurrent paleogeographic and climatic events.

Consistent with the *F*_*ST*_ analysis results and established genetic differentiation thresholds (*F*_*ST*_ < 0.05 denotes low genetic differentiation; *F*_*ST*_ > 0.25 indicates significant population divergence) [39], our findings reveal moderate to substantial genetic divergence between CB and the other two populations (*F*_*ST*_: 0.0817701–0.185934). This range far exceeds the low-differentiation threshold. In contrast, low levels of genetic differentiation are observed between CM and CH, with their* F*_*ST*_ values (0.0420637∼0.0527671) mostly falling below or marginally approaching the 0.05 threshold.

Taking into account the combined evidence of substantial genetic differentiation between CB and the other two populations, together with the absence of diagnostic morphological differences across all three, we tentatively conclude that *C. bouderius* is likely the sole valid species within the genus *Cranoglanis*. However, several limitations of this study should be acknowledged. Although we conducted comparative analyses of meristic traits among the three groups, we did not perform more detailed morphological reassessments, including morphometric measurements, meristic counts, or geometric morphometric analyses. Moreover, niche divergence and niche conservatism serve as core criteria for determining whether genetically distinct populations ought to be designated as species, ecotypes, or evolutionarily significant units (ESUs) [40, 41]. The ecological context underlying the divergence of these populations was not examined in the present study. Therefore, future research should incorporate ecological niche modeling or comparative habitat analyses using environmental data from the Pearl River, Hainan Island, and Red River systems. In summary, additional morphological data and a comprehensive understanding of the ecological factors driving population divergence are necessary to further substantiate our conclusions.

### Genetic diversity and effective population size

Genetic diversity forms the foundation for species' evolutionary adaptation to environmental changes and serves as a key indicator for evaluating the status of biological resources; its reduction poses substantial threats to the sustainability of wild populations [42]. Prior studies on the genetic diversity of *Cranoglanis* in China have been restricted to mitochondrial markers [6, 7, 43]; this study, however, is the first to assess genetic variation in these populations using genome-wide single nucleotide polymorphisms (SNPs). As a product of long-term evolution, genetic diversity is also a prerequisite for species’ survival, adaptation, and evolutionary development [44]. Notably, we estimated the average nucleotide diversity across three *Cranoglanis* populations, marking the first report of genetic variation in helmet catfish based on genome-wide SNPs. Our analysis showed that the CB population had the lowest nucleotide diversity (π = 0.0689–0.0723), in contrast to CM (π = 0.2392) and CH (π = 0.2922–0.2964). This finding contradicts earlier mitochondrial marker-based studies, which reported CM as the population with the lowest genetic diversity [6, 7].

To resolve this discrepancy, we further analyzed two mitochondrial markers (Cytb and COI). Paradoxically, these markers revealed CB had the highest diversity (Cytb π = 0.00340; COI π = 0.00231), CM the lowest (Cytb π = 0.00075; COI π = 0.00053), and CH intermediate values (Table S5). Notably, the nucleotide diversity of CH (Cytb π = 0.00330; COI π = 0.00181) was closely aligned with that of CB. Clearly, the genetic diversity patterns inferred from mitochondrial markers were inconsistent with those derived from genome-wide SNPs in the present study. The genetic diversity and population structure inferred from mtDNA and genome-wide SNPs were inconsistent a common phenomenon in fish and other animal populations. The discrepancies between the two primarily stem from inherent differences in genetic information content, evolutionary dynamics, and statistical power [44]. Similar discrepancies have been observed in other species, such as the montane caddisfly *Thremma gallicum*, where RAD data and mtDNA produced conflicting phylogeographic signals [45]. mtDNA is a single maternally inherited marker with a smaller effective population size than the nuclear genome. It does not undergo recombination, making it highly susceptible to genetic drift, historical bottlenecks, and the randomness of maternal lineages. Furthermore, mtDNA is more likely to be affected by selective events (e.g., adaptive selection related to energy metabolism) or nuclear pseudogenes (numts) [46, 47]. These factors result in a genetic pattern that cannot fully represent the history and structure of the entire genome. In contrast, genome-wide SNPs are based on tens of thousands of independent nuclear loci, incorporating genetic information from both parents [48, 49]. They offer higher statistical power, enabling more accurate and stable reflections of overall genetic diversity, population differentiation, and gene flow patterns. The integrated multi-locus signals can "average out" biases caused by selection or random drift at individual loci, thus better revealing the true population structure and evolutionary history [50]. Particularly in the presence of sex-biased dispersal, historical hybridization, or local selection pressures, mtDNA and the nuclear genome tend to show significant discrepancies—whereas genome-wide SNPs are more capable of capturing the overall genetic background at the population level [51, 52].

Therefore, the inconsistency between the two sets of results is not contradictory but rather reflects their respective genetic characteristics. mtDNA primarily reflects the unique history of maternal lineages, while genome-wide SNPs reveal the overall genetic structure of the species from a broader nuclear genetic perspective. Due to their high resolution and ability to integrate diverse evolutionary processes, the genome-wide SNP results serve as the primary basis in this study for interpreting the genetic diversity and population relationships of *Cranoglanis*. Meanwhile, the mtDNA differences are treated as supplementary clues to indicate potential maternal historical features or local selection signals. In conclusion, our genome-wide SNP findings revealing the lowest diversity in CB and the highest in CH likely provide the most accurate representation of contemporary genetic variation patterns among *Cranoglanis* populations.

To further validate these genetic diversity patterns, we analyzed individual heterozygosity based on 326,322 genotyped sites, which uncovered distinct population-specific patterns among CB, CM, and CH. The CB population exhibited the highest mean heterozygosity index (0.7072) with a relatively narrow range (0.6401–0.7305), indicating consistent and high heterozygosity across individuals. In contrast, the CM population (H-prefixed individuals) showed a moderate mean heterozygosity index (−0.0048) but substantial inter-individual variation (range: −0.5602 to 0.1115). The CH population had the lowest mean heterozygosity index (−0.1211) with a narrow range (−0.1949 to −0.0613), reflecting uniformly reduced individual heterozygosity.

To complement this individual-level assessment, we further analyzed observed heterozygosity (ObsHet) and expected heterozygosity (ExpHet), which were consistent between variant positions and all positions across all populations. The seven subpopulations fell into three distinct genetic diversity groups: RD, RL, and RR exhibited high ObsHet (0.2963–0.2988), HN and RY showed moderate ObsHet (0.2647–0.2883), while ZH and ZL (CB subpopulations) had extremely low ObsHet (0.0768–0.078) (Tab. S6). For each population, ObsHet was slightly higher than ExpHet, indicating no significant deviation from Hardy–Weinberg equilibrium. Notably, these heterozygosity patterns were consistent with our π results, collectively confirming drastic genetic diversity differentiation among the populations.

Our linkage disequilibrium (LD) analysis further supported the genetic diversity patterns. LD, representing non-random allele association across loci, is shaped by recombination rates, demographic history, selection pressures, and genetic drift [53]. The observed LD decay patterns revealed distinct population characteristics: CM exhibited the slowest decay rate when r^2^ reached the 0.1 threshold, while CB reached the baseline value first, aligning with expectations that higher genetic diversity corresponds to shorter LD decay distances [53]. This observation parallels established patterns where wild populations typically show low LD due to diverse gene pools, contrasting with domesticated populations that demonstrate extended LD resulting from selective breeding [54]. The CH population, displaying the highest genetic diversity, correspondingly showed the most rapid LD decay rate. Notably, CB and CM populations showed divergent LD patterns depending on evaluation metrics, with baseline value comparisons providing results most consistent with our genetic diversity assessments.

To investigate historical demographic dynamics, we performed PSMC analysis, which revealed coordinated patterns across all three *Cranoglanis* populations: N_e_ began declining approximately 1 Mya and reached minimum values around 0.2 Mya. This period coincides with the Pleistocene Glaciations (2.6 million—12,000 years ago), marked by cyclical glacial advances that induced global cooling and substantially impacted tropical/subtropical freshwater ecosystems [55, 56]. This climatic shift may have led to the continuous reduction in the size of the three populations. The population minima at ~ 0.2 Mya align temporally with peak cooling during the Last Glacial Period's initial phase [57]. Subsequent N_e_ recovery slowly from 0.2 Mya to 50 thousand years ago (ka) corresponds to the Last Interglacial period, when warmer-than-present conditions [58]. This warmer climate likely enhanced freshwater ecosystems in tropical and subtropical regions, creating more favorable habitats and supporting the recovery and expansion of the CB, CH, and CM populations [56].

Notably, CH and CM populations began to decline around 50 ka, likely due to the fact that this period coincided with the middle phase of the Last Glacial Period, characterized by global cooling, glacial expansion, and a significant drop in sea levels [55]. As CH and CM are located in coastal regions, they were more vulnerable to the impacts of sea-level changes, which likely led to habitat loss and fragmentation. In contrast, the CB population, situated inland Pearl River Basin, remained relatively insulated from these marine influences. In fact, the lowered sea levels may have enhanced wetland and river habitats in this region, providing expanded ecological resources that supported continued population growth. The situation was markedly different for CH and CM, where the scarcity of suitable climate refuges in Vietnam and Hainan likely compounded their environmental stresses during this harsh climatic period [57, 59].

While PSMC uncovered historical demographic fluctuations associated with climatic events, analyses of recent N_e_ via GONE demonstrated long-term stability across all three populations over recent generations. The GONE results showed no significant N_e_ fluctuations, expansions, or contractions over the observed 50–750 generations; instead, all three maintained stable N_e_ levels with a clear hierarchical order: CH retained the largest N_e_ (1 × 10⁶–1 × 10⁷), followed by CB (1 × 10^5^–1 × 10⁶) and CM (1 × 10^4^–1 × 10^5^). This stability suggests that the historical climatic pressures (e.g., glaciations, sea-level changes) that drove N_e_ fluctuations have diminished in the recent period, and contemporary factors (e.g., stable habitat availability, balanced birth/death rates, and limited anthropogenic disturbance) now sustain population sizes.

Notably, our GONE-derived recent N_e_ estimates reveal a key discrepancy that merits clarification. CM exhibits the smallest recent N_e_ (1 × 10^4^–1 × 10^5^) and the lowest mtDNA π, yet its genome-wide SNP π (0.2392) is comparable to that of CH (0.2922–0.2964). Conversely, CB possesses a higher recent N_e_ (1 × 10^5^–1 × 10⁶) but the lowest SNP π (0.0689–0.0723). This apparent mismatch may be partially accounted for by three interrelated factors, which are supported in part by our comprehensive genetic, demographic, and individual heterozygosity data. First, nuclear genes are biparentally inherited, so male-mediated dispersal can facilitate genetic exchange, boosting CM's SNP π without necessarily altering its recent Nₑ. In contrast, mtDNA is maternally inherited, and limited female dispersal prevents such supplementation, leaving CM's mtDNA π as the lowest observed (Cytb: 0.00075; COI: 0.00053). For CB, despite its higher recent Nₑ and high mean heterozygosity (0.7072, range: 0.6401–0.7305) resulting from ZH/ZL subpopulation mixing, its low SNP π likely arises from inefficient asymmetric CH → CB gene flow (Nₘ ≈ 1.03). This level of gene flow may be insufficient to offset long-term diversity loss from historical bottlenecks and habitat disturbances.

Second, differential selection and population history further shape this pattern. mtDNA, which encodes core energy metabolism genes, is under strong purifying selection. CM's isolated Red River Basin habitat may intensify this selection, eliminating harmful mutations and reducing mtDNA π. Genome-wide SNPs, however, include numerous neutral or near-neutral loci (e.g., synonymous mutations, introns) that are less affected by selection, thus preserving CM's historical nuclear diversity. For CB, LD analysis shows it reaches baseline r^2^ most rapidly, consistent with low SNP π resulting from long-term habitat disturbances and genetic drift. Its high heterozygosity likely reflects recent subpopulation mixing rather than long-term mutation accumulation, which may explain the decoupling between heterozygosity and SNP π. It should be noted that these inferences are based on existing research data, and further studies will be necessary to validate these propositions.

In summary, the CH population maintains the largest recent effective size (Nₑ, 1 × 10⁶–1 × 10⁷), while CB's Nₑ (1 × 10^5^–1 × 10⁶) is already lower. Notably, CB also exhibits significantly lower genetic diversity than CH. This dual disadvantage, consisting of both a subordinate recent Nₑ and reduced genetic diversity, highlights an urgent need for targeted conservation measures to safeguard the long-term viability of the Pearl River CB population.

### Genetic adaptations caused by long-term isolation among the three populations

The KEGG enrichment analysis revealed significant differences in biological pathways among the three geographically isolated fish populations: CB (Pearl River Basin), CM (Hainan Island), and CH (Red River Basin, Vietnam). These differences likely reflect their prolonged adaptation to distinct environmental conditions and selective pressures, driven by their unique ecological contexts. Complementing these functional insights, the Manhattan plots provide a genome-scale perspective of genetic differentiation, with Z-transformed *F*_*ST*_ values highlighting genomic regions under putative selection. These results align closely with the pathway enrichment findings.

The enrichment of viral-related pathways, such as "viral protein interaction with cytokine and cytokine receptor" and "viral carcinogenesis," in comparisons like CB_VS_CH_CB and CM_VS_CB_CB, suggests that these populations may have evolved distinct antiviral mechanisms due to varying viral exposure in their respective habitats. The Pearl River Basin (CB) and Red River Basin (CH), characterized by their complex aquatic environments, may harbor a higher diversity of viruses, leading to stronger selection pressures on immune-related pathways. In contrast, the relatively isolated environment of Hainan Island (CM) may result in lower viral diversity and thus less pronounced selection on antiviral mechanisms [60, 61].

Immune-related pathways, including "cytokine-cytokine receptor interaction" and "chemokine signaling pathway," were significantly enriched in multiple comparisons, indicating that the three populations may have developed unique immune adaptations. The CB and CH populations, inhabiting more complex and potentially pathogen-rich environments, likely exhibit enhanced immune responses compared to the CM population, which may face fewer pathogens due to its isolated habitat [62, 63].

The enrichment of metabolic pathways, such as "purine metabolism" and "starch and sucrose metabolism," in CM_VS_CH_CM suggests that the CM population may have adapted to a more resource-limited environment. Hainan Island's relatively isolated and potentially nutrient-poor waters could have driven the evolution of energy-efficient metabolic strategies in the CM population. In contrast, the CB and CH populations, inhabiting nutrient-rich river basins, may have evolved metabolic pathways optimized for high-energy utilization [64].

The significant enrichment of nervous system-related pathways, such as "GABAergic synapse" and "cholinergic synapse," in CM_VS_CH_CM may reflect adaptations in behavior and sensory perception. The isolated environment of Hainan Island could have led to unique evolutionary pressures on the nervous system, potentially influencing social interactions, foraging behavior, or environmental sensing in the CM population [65, 66].

In conclusion, the observed differences in pathway enrichment among the three populations highlight the role of long-term geographic isolation and environmental adaptation in shaping their genetic and functional diversity. These findings provide valuable insights into the evolutionary dynamics of geographically isolated populations and underscore the importance of considering environmental factors in studies of adaptive evolution.

## Conclusions

The study investigated the genetic and functional diversity of three geographically isolated populations of *Cranoglanis* fish: CB (Pearl River Basin), CM (Hainan Island), and CH (Red River Basin, Vietnam). Morphological and genetic analyses revealed no significant differences in countable traits among the populations, but genomic data indicated distinct population structures. The CM and CH populations showed closer genetic relationships, likely due to historical connections via the Red River Fault Zone, while CB exhibited greater genetic differentiation. Genetic diversity analyses based on genome-wide SNPs revealed that CB had the lowest diversity, whereas CH had the highest, contrasting with previous mitochondrial marker studies. Effective population size (N_e_) estimates suggested that all populations experienced declines during the Pleistocene glaciations, with CB recovering more robustly due to its inland location. However, recent N_e_ inference via GONE software (covering 50–750 generations) revealed a reversed pattern: the CH population now maintains a larger effective size than CB. KEGG enrichment analysis highlighted adaptations in viral and immune-related pathways in CB and CH, likely due to higher pathogen pressures, while CM showed adaptations in metabolic and nervous system pathways, reflecting its resource-limited and isolated environment. These findings underscore the impact of long-term geographic isolation and environmental pressures on the genetic and functional divergence of these populations, emphasizing the need for targeted conservation efforts, particularly for the CB population, which shows lower genetic diversity despite its larger effective population size.

## Supplementary Information

Supplementary Material 1.

Supplementary Material 2.

Supplementary Material 3.

## Data Availability

No datasets were generated or analysed during the current study.

## Abbreviations

AIC: Akaike Information Criterion
AFLP: Amplified Fragment Length Polymorphism
AM: Ancient Migration
BAM: Binary Alignment Map
BLAST: Basic Local Alignment Search Tool
bp: Base pair
CB: *Cranoglanis**bouderius* (Pearl River population)
CH: *Cranoglanis**henrici* (Red River population)
CM: *Cranoglanis**multiradiatus* (Hainan Island population)
COI: Cytochrome c oxidase subunit I
cPAS: Combinatorial Probe-Anchor Synthesis
Cytb: Cytochrome b
D-loop: Displacement loop (mitochondrial DNA)
DNB: DNA Nanoball
DNA: Deoxyribonucleic Acid
ESU: Evolutionarily Significant Unit
*F*_*ST*_: Fixation Index (population differentiation)
GATK: Genome Analysis Toolkit
GO: Gene Ontology
GONE: Software for estimating recent effective population size
IACUC: Institutional Animal Care and Use Committee
IM: Isolation-with-Migration
ka: Thousand years ago
KEGG: Kyoto Encyclopedia of Genes and Genomes
LD: Linkage Disequilibrium
MAF: Minor Allele Frequency
ML: Maximum Likelihood
MIS: Marine Isotope Stage
mtDNA: Mitochondrial DNA
Mya: Million years ago
N_e_: Effective Population Size
NGS: Next-Generation Sequencing
Nm: Effective number of migrants per generation
NR: Non-Redundant protein database
ObsHet: Observed Heterozygosity
PCA: Principal Component Analysis
PCR: Polymerase Chain Reaction
PSMC: Pairwise Sequentially Markovian Coalescent
QC: Quality Control
RAD-seq: Restriction site-Associated DNA sequencing
RAxML: Randomized Axelerated Maximum Likelihood
SC: Secondary Contact
SDS: Sodium Dodecyl Sulfate
SFS: Site Frequency Spectrum
SI: Strict Isolation
SNP: Single Nucleotide Polymorphism
ssCirDNA: Single-strand Circular DNA
TE buffer: Tris–EDTA buffer
UniProtKB: Universal Protein Knowledgebase
VCF: Variant Call Format
ya: Years ago

## References

[1] Peng Z, Wang J, He S. The complete mitochondrial genome of the helmet catfish *Cranoglanis bouderius* (Siluriformes: Cranoglanididae) and the phylogeny of otophysan fishes. Gene. 2006;376:290–7. 16737786 10.1016/j.gene.2006.04.014

[2] Xie S, Zhou A, Feng Y, Wang Z, Fan L, Zhang Y, et al. Effects of fasting and re-feeding on mstn and mstnb genes expressions in *Cranoglanis bouderius*. Gene. 2019;682:1–12. 30267811 10.1016/j.gene.2018.09.050

[3] Ng HH, Kottelat M. *Cranoglanis henrici* (Vaillant, 1893), a valid species of cranoglanidid catfish from Indochina (Teleostei, Cranoglanididae). Zoosystema. 2000;22:847–52.

[4] Liu C, Peng Z, He S. Studies on species classification for genus *Cranoglanis* Peters with the method of morphometrics. Acta Hydrobiol Sin. 2005;29:512.

[5] Cheng F, Xie SG, Wei YE, Fu-Liang YE. Genetic analysis of *Cranoglanis**bouderius* by molecular marker AFLP. Acta Hydrobiol Sin. 2009;33:539–45.

[6] Shaolin X, Chao W, Zijun L, Zhengguang LI, Rennai S, Jixing Z. Analysis of population genetic structure of *Cranoglanis* based on mitochondrial Dloop sequences. Journal of South China Agricultural University. 2016;37(1):8-13. 10.7671/j.issn.1001-411X.2016.01.002.

[7] Shaolin X, Zijun LV, Aiguo Z, Jintao C, Zhengguang LI, Jixing Z. Population genetic structure of *C. multiradiatus*, *C. bouderius* and *C. henrici* on the mitochondrial Cytb and COⅠsequence. Ecol Sci. 2016;35(3):65–72.

[8] Sung W, Peiqi Y, Yiyu C. China Red Data Book of Endangered Animals: Pisces. Beijing: Science Press;1998.

[9] Jiang Z, Jiang J, Wang Y, Zhang E, Zhang Y, Li L, et al. Red list of China’s vertebrates. Biodiversity Science. 2016;24:500–51.

[10] Gao ZY, Zhang Q, Xia YH, Chen DY, Zhu Y, Gui-Sheng LI. Genetic diversity of *Cranoglanis bouderius* in Songtao reservoir. Guangdong Agricultural Sciences. 2013;40(3):98–100.

[11] Yue XH, Zhao S, Liu HL. Genetic variation of *Cranoglanis bouderius multiradiatus* population in Nandujiang River base on mitochondrial cytochrome b sequences. Ecological Science. 2010;29(03):247–50.

[12] Wang J, Xue DX, Zhang BD, Li YL, Liu BJ, Liu JX. Genome-wide SNP discovery, genotyping and their preliminary applications for population genetic inference in spotted sea bass (*Lateolabrax maculatus*). PLoS ONE. 2016;11:e0157809. 27336696 10.1371/journal.pone.0157809 PMC4919078

[13] Maroso F, Gkagkavouzis K, De Innocentiis S, Hillen J, do Prado F, Karaiskou N, et al. Genome-wide analysis clarifies the population genetic structure of wild gilthead sea bream (*Sparus**aurata*). PLoS ONE. 2021;16:e0236230. 33428622 10.1371/journal.pone.0236230 PMC7799848

[14] Caixia L, Zuogang P, Shunping H. Studies on species classification for genus Cranoglanis Peters with the method of morphometrics. Acta Hydrobiologica Sinica. 2005;29.

[15] Chen Y, Chen Y, Shi C, Huang Z, Zhang Y, Li S, et al. SOAPnuke: a MapReduce acceleration-supported software for integrated quality control and preprocessing of high-throughput sequencing data. Gigascience. 2018;7:1–6. 29220494 10.1093/gigascience/gix120 PMC5788068

[16] Li H, Durbin R. Fast and accurate short read alignment with Burrows-Wheeler transform. Bioinformatics. 2009;25:1754–60. 19451168 10.1093/bioinformatics/btp324 PMC2705234

[17] Li H, Handsaker B, Wysoker A, Fennell T, Ruan J, Homer N, et al. The Sequence Alignment/Map format and SAMtools. Bioinformatics. 2009;25:2078–9. 19505943 10.1093/bioinformatics/btp352 PMC2723002

[18] Danecek P, Auton A, Abecasis G, Albers CA, Banks E, DePristo MA, et al. The variant call format and VCFtools. Bioinformatics. 2011;27:2156–8. 21653522 10.1093/bioinformatics/btr330 PMC3137218

[19] Catchen J, Hohenlohe PA, Bassham S, Amores A, Cresko WA. Stacks: an analysis tool set for population genomics. Mol Ecol. 2013;22:3124–40. 23701397 10.1111/mec.12354 PMC3936987

[20] Hu Y, Li H, Xia J, Li C. Population structure, genetic diversity, and conservation strategies of a commercially important sleeper fish, *Odontobutis potamophilus* (Gobiiformes: Odontobutidae) based on gene-capture data. Front Genet. 2022;13:843848. 35685434 10.3389/fgene.2022.843848 PMC9171042

[21] Stamatakis A. RAxML version 8: a tool for phylogenetic analysis and post-analysis of large phylogenies. Bioinformatics. 2014;30(9):1312–3. 24451623 10.1093/bioinformatics/btu033 PMC3998144

[22] Pritchard JK, Stephens MF, Donnelly P. Inference of population structure using multilocus genotype data. Genetics. 2000;155(2):945–59. 10835412 10.1093/genetics/155.2.945 PMC1461096

[23] Earl DA, vonHoldt BM. STRUCTURE HARVESTER: a website and program for visualizing STRUCTURE output and implementing the Evanno method. Conserv Genet Resour. 2011;4:359–61.

[24] Excoffier LA-O, Marchi N, Marques DA, Matthey-Doret R, Gouy A, Sousa VC. Fastsimcoal2: demographic inference under complex evolutionary scenarios. Bioinformatics. 2021;37:4882–5. 34164653 10.1093/bioinformatics/btab468 PMC8665742

[25] Zhang C, Dong SS, Xu JY, He WM, Yang TL. PopLDdecay: a fast and effective tool for linkage disequilibrium decay analysis based on variant call format files. Bioinformatics. 2019;35:1786–8. 30321304 10.1093/bioinformatics/bty875

[26] Zhao S, Zheng P, Dong S, Zhan X, Wu Q, Guo X, et al. Whole-genome sequencing of giant pandas provides insights into demographic history and local adaptation. Nat Genet. 2012;45:67–71. 23242367 10.1038/ng.2494

[27] Gao J, Xu G, Xu P. Whole-genome resequencing of three *Coilia nasus* population reveals genetic variations in genes related to immune, vision, migration, and osmoregulation. BMC Genomics. 2021;22:878. 34872488 10.1186/s12864-021-08182-0 PMC8647404

[28] Wright S. Evolution in Mendelian Populations. Genetics. 1931;16(2):97–159. 10.1093/genetics/16.2.97. 10.1093/genetics/16.2.97 PMC1201091 17246615

[29] Luckenbill KR, Lundberg JG. CAT scan-based images of the skeleton of the Asian Catfish (Siluriformes: Cranoglanididae). P Acad Nat Sci Phila. 2009;158:297–9.

[30] Liu C. Studies on Systematics and Species Validity of the Genera Cranoglanis (Silurformes: Cranoglanididae) and Coilia (Clupeiformes: Engraulidae) [master]: Institute of Hydrobiology, Chinese Academy of Sciences; 2007.

[31] Liang G. Eight evidences about Hainan Island separated from Chinas Beibuwan Gulf with drifting and rotation. Acta Geol Sin. 2013;87:73–6.

[32] Mo Y, Shi Y. Paleomagnetic study and tectonic evolution of Hainan terrane and its vicinal continental coast the late Mesozoic to Cenozoic. J Nanjing Univ. 1987;23(3):521–32.

[33] Zhu H. Biogeographical evidences help revealing the origin of Hainan Island. PLoS ONE. 2016;11:e0151941. 27055236 10.1371/journal.pone.0151941 PMC4824349

[34] Jiaqi C, Chao LI, Wenjun Z, Wei LI, Tianyang G, Jun Z. Genetic variation and phylogeography of *Channa gachua* in Hainan Island. Acta Ecol Sin. 2019;39:2591–602.

[35] Lin LH, Ji X, Diong CH, Du Y, Lin CX. Phylogeography and population structure of the Reevese’s Butterfly Lizard (*Leiolepis reevesii*) inferred from mitochondrial DNA sequences. Mol Phylogenet Evol. 2010;56:601–7. 20433932 10.1016/j.ympev.2010.04.032

[36] Yan TU, Wenshen X, Rujian W, Renhui XU. Sediment provenance variations in the Canada Basin, western Arctic Ocean since MIS 8: implications on Arctic ice sheet and circulation changes. Quaternary SciencesVL. 2021;41:632–45.

[37] Chen Y, Hu Y, Jiang X, Li J, Shang Z, Fang J, et al. Time correlation between MIS5a transgression and global sea level change of the second Marine layer in the coastal lowland of Bohai Bay. Geology in China. 2024;51:2056–65.

[38] Kaufman D, McKay N, Routson C, Erb M, Dätwyler C, Sommer PS, et al. Holocene global mean surface temperature, a multi-method reconstruction approach. Sci Data. 2020;7:201. 32606396 10.1038/s41597-020-0530-7 PMC7327079

[39] Wright SD. Evolution and the Genetics of Populations, Volume 4: Variability Within and Among Natural Populations. Chicago: University of Chicago Press; 1978.

[40] Holt RD. Bringing the Hutchinsonian niche into the 21st century: ecological and evolutionary perspectives. Proc Natl Acad Sci U S A. 2009;106 Suppl 2(Suppl 2):19659–65. 19903876 10.1073/pnas.0905137106 PMC2780934

[41] McCormack JE, Zellmer AJ, Knowles LL. Does niche divergence accompany allopatric divergence in Aphelocoma jays as predicted under ecological speciation? Insights from tests with niche models. Evolution. 2010;64(5):1231–44. 19922442 10.1111/j.1558-5646.2009.00900.x

[42] Manel S, Guerin P-E, Mouillot D, Blanchet S, Velez L, Albouy C, et al. Global determinants of freshwater and marine fish genetic diversity. Nat Commun. 2020;11:692. 32041961 10.1038/s41467-020-14409-7 PMC7010757

[43] Zhi-Yuan G, Qun Z, Yue-Heng X, De-Yin C, Ye Z, Gui-Sheng LI. Genetic diversity of *Cranoglanis bouderius* in Songtao reservoir. Guangdong Agricultural Sciences. 2013.

[44] Liu J, You X, Xu P, Zhuang P, Zheng Y, Zhang K, et al. Assessing the genetic diversity of the critically endangered Chinese sturgeon *Acipenser sinensis* using mitochondrial markers and genome-wide single-nucleotide polymorphisms from RAD-seq. Sci China Life Sci. 2018;61:1090–8. 29948902 10.1007/s11427-017-9254-6

[45] Macher JN, Rozenberg A, Pauls SU, Tollrian R, Wagner R, Leese F. Assessing the phylogeographic history of the montane caddisfly *Thremma gallicum* using mitochondrial and restriction‐site‐associated DNA (RAD) markers. Ecol Evol. 2015;5:648–62. 25691988 10.1002/ece3.1366 PMC4328769

[46] Ballard JWO, Dean MD. The mitochondrial genome: mutation, selection and recombination. Curr Opin Genet Dev. 2001;11:667–72. 11682311 10.1016/s0959-437x(00)00251-3

[47] Kremer LS, Golder Z, Barton-Owen T, Papadea P, Koolmeister C, Chinnery PF, et al. The bottleneck for maternal transmission of mtDNA is linked to purifying selection by autophagy. Sci Adv. 2025;11:eaea4660. 41223274 10.1126/sciadv.aea4660 PMC12609065

[48] Allendorf FW, Hohenlohe PA, Luikart G. Genomics and the future of conservation genetics. Nat Rev Genet. 2010;11(10):697–709. 20847747 10.1038/nrg2844

[49] Davey JW, Hohenlohe PA, Etter PD, Boone JQ, Catchen JM, Blaxter ML. Genome-wide genetic marker discovery and genotyping using next-generation sequencing. Nat Rev Genet. 2011;12(7):499–510. 21681211 10.1038/nrg3012

[50] Zhang L, Li H, Shi M, Ren K, Zhang W, Cheng Y, et al. FishSNP: a high quality cross-species SNP database of fishes. Sci Data. 2024;11(1):286. 38461307 10.1038/s41597-024-03111-8 PMC10924876

[51] Bonnet T, Leblois R, Rousset F, Crochet PA. A reassessment of explanations for discordant introgressions of mitochondrial and nuclear genomes. Evolution. 2017;71(9):2140–58. 28703292 10.1111/evo.13296

[52] Runemark AA-O, Eroukhmanoff FA-O, Nava-Bolaños A, Hermansen JA-O, Meier JI. Hybridization, sex-specific genomic architecture and local adaptation. Philos Trans R Soc Lond B Biol Sci. 2018;373(1757):20170419. 10.1098/rstb.2017.0419. 30150218 10.1098/rstb.2017.0419 PMC6125728

[53] Slatkin M. Linkage disequilibrium–understanding the evolutionary past and mapping the medical future. Nat Rev Genet. 2008;9(6):477–85. 18427557 10.1038/nrg2361 PMC5124487

[54] Zheng J, Zhao L, Zhao X, Gao T, Song N. High genetic connectivity inferred from whole-genome resequencing provides insight into the phylogeographic pattern of *Larimichthys polyactis*. Mar Biotechnol. 2022;24:671–80. 10.1007/s10126-022-10134-y 35701688

[55] Clark PU, Dyke AS, Shakun JD, Carlson AE, Clark J, Wohlfarth B, Mitrovica JX, et al. The last glacial maximum. Science. 2009;325(5941):710–4. 19661421 10.1126/science.1172873

[56] Hewitt G. The genetic legacy of the Quaternary ice ages. Nature. 2000;405(6789):907–13. 10879524 10.1038/35016000

[57] Provan J, Bennett KD. Phylogeographic insights into cryptic glacial refugia. Trends Ecol Evol. 2008;23(10):564–71. 18722689 10.1016/j.tree.2008.06.010

[58] Otto-Bliesner BL, Marshall SJ, Overpeck JT, Miller GH, Hu A. Simulating Arctic climate warmth and icefield retreat in the last interglaciation. Science. 2006;311(5768):1751–3. 16556838 10.1126/science.1120808

[59] Lambeck K, Chappell J. Sea level change through the last glacial cycle. Science. 2001;292(5517):679–86. 11326090 10.1126/science.1059549

[60] Suttle CA. Marine viruses--major players in the global ecosystem. Nat Rev Microbiol. 2007;5(10):801–12. 17853907 10.1038/nrmicro1750

[61] Vignuzzi M, López CA. Defective viral genomes are key drivers of the virus-host interaction. Nat Microbiol. 2019;4(7):1075–87. 31160826 10.1038/s41564-019-0465-y PMC7097797

[62] Piertney SB, Oliver MK. The evolutionary ecology of the major histocompatibility complex. Heredity (Edinb). 2006;96(1):7–21. 16094301 10.1038/sj.hdy.6800724

[63] Schulenburg H, Kurtz J, Moret Y, Siva-Jothy MT. Introduction. Ecological immunology. Philos Trans R Soc Lond B Biol Sci. 2009;364(1513):3–14. 18926970 10.1098/rstb.2008.0249 PMC2666701

[64] Bozinovic F, Pörtner HO. Physiological ecology meets climate change. Ecol Evol. 2015;5(5):1025–30. 25798220 10.1002/ece3.1403 PMC4364817

[65] Niven JE, Laughlin SB. Energy limitation as a selective pressure on the evolution of sensory systems. J Exp Biol. 2008;211(Pt 11):1792–804. 18490395 10.1242/jeb.017574

[66] Losos JB, Ricklefs RE. Adaptation and diversification on islands. Nature. 2009;457(7231):830–6. 19212401 10.1038/nature07893

