# Supplementary material for: Whole-genome resequencing reveals genetic divergence, local adaptation, and conservation priorities in three helmet catfishes (complex Cranoglanis bouderius)
Source: BMC Genomics. 2026 Apr 14;27:500. doi: 10.1186/s12864-026-12843-3 (PMC13188337; doi:10.1186/s12864-026-12843-3)
Supplement: Supplementary file 2 — Supplementary Material 2. [file 12864_2026_12843_MOESM2_ESM.docx]

**Whole-genome resequencing reveals genetic divergence, local adaptation, and conservation priorities in three helmet catfishes (complex** ***Cranoglanis bouderius*)**

Shaolin Xie^1#^, Yun Hu^2,3#^, Jiantao Hu^2,3#^, Dongjie Wang^1^, Aiguo Zhou^1^, Yusen Li^5^, Bo Huang^5^, Vanthu Giap^2,3^, Tuan Anh Trieu^6^, Chenhao Zou^4^*, Chenhong Li^2,3^**

^1^College of Marine Sciences, South China Agricultural University, Guangzhou, Guangdong, 510642, China

^2^ Shanghai Universities Key Laboratory of Marine Animal Taxonomy and Evolution, Shanghai Ocean University, Shanghai 201306, China;

^3^Engineering Research Center of Environmental DNA and Ecological Water Health Assessment, Shanghai Ocean University, Shanghai 201306, China

^4^ Department of Computer Science, Lakehead University, Thunder Bay, Ontario P7B 5E1, Canada

^5^ Engineering Research Center of Hongshui River Rare Fish Conservation, Guangxi Zhuang Autonomous Region, Guangxi Academy of Fishery Sciences, Nanning 530021, China

^6^ Faculty of Natural Sciences, Hung Vuong University, Nong Trang, Viet Tri, Phu Tho, Vietnam.

^#^These authors contributed equally to this work.

**^*^Corresponding author**:* Chenhao Zou, Department of Computer Science, Lakehead University, Thunder Bay, Ontario P7B 5E1, Canada, E-mail:chenhaozouprivate@gmail.com;** Chenhong Li, Shanghai Universities Key Laboratory of Marine Animal Taxonomy and Evolution, Shanghai Ocean University, Shanghai 201306, China; Email: chli@shou.edu.cn;

Table S1 Clean reads per individual

| Sample Name | Clean Reads | Clean Base | Read Length |
| --- | --- | --- | --- |
| ZL1 | 76,540,958 | 22,962,287,400 | 150 |
| ZL2 | 70,347,447 | 21,104,234,100 | 150 |
| ZL3 | 73,722,826 | 22,116,847,800 | 150 |
| ZL4 | 74,133,987 | 22,240,196,100 | 150 |
| ZL5 | 74,343,043 | 22,302,912,900 | 150 |
| ZL6 | 79,389,926 | 23,816,977,800 | 150 |
| ZL7 | 79,224,969 | 23,767,490,700 | 150 |
| ZL8 | 69,270,457 | 20,781,137,100 | 150 |
| ZL9 | 94,583,905 | 28,375,171,500 | 150 |
| ZL10 | 81,183,249 | 24,354,974,700 | 150 |
| ZH1 | 75,038,988 | 22,511,696,400 | 150 |
| ZH2 | 69,105,512 | 20,731,653,600 | 150 |
| ZH3 | 75,753,972 | 22,726,191,600 | 150 |
| ZH4 | 80,373,453 | 24,112,035,900 | 150 |
| ZH5 | 76,632,395 | 22,989,718,500 | 150 |
| ZH6 | 80,596,202 | 24,178,860,600 | 150 |
| ZH7 | 100,089,764 | 30,026,929,200 | 150 |
| H1 | 79,957,609 | 23,987,282,700 | 150 |
| H2 | 66,734,764 | 20,020,429,200 | 150 |
| H3 | 78,124,078 | 23,437,223,400 | 150 |
| H4 | 73,232,007 | 21,969,602,100 | 150 |
| H5 | 82,661,288 | 24,798,386,400 | 150 |
| H6 | 91,407,081 | 27,422,124,300 | 150 |
| H7 | 78,942,062 | 23,682,618,600 | 150 |
| H8 | 76,744,548 | 23,023,364,400 | 150 |
| H9 | 74,865,841 | 22,459,752,300 | 150 |
| H10 | 86,795,557 | 26,038,667,100 | 150 |
| H11 | 74,973,456 | 22,492,036,800 | 150 |
| H12 | 72,741,361 | 21,822,408,300 | 150 |
| H13 | 104,082,244 | 31,224,673,200 | 150 |
| H14 | 69,094,522 | 20,728,356,600 | 150 |
| H15 | 73,639,209 | 22,091,762,700 | 150 |
| H16 | 95,662,299 | 28,698,689,700 | 150 |
| H17 | 71,889,408 | 21,566,822,400 | 150 |
| H18 | 73,907,369 | 22,172,210,700 | 150 |
| H19 | 71,562,513 | 21,468,753,900 | 150 |
| H20 | 69,742,680 | 20,922,804,000 | 150 |
| RD1 | 71,764,907 | 21,529,472,100 | 150 |
| RD2 | 80,824,293 | 24,247,287,900 | 150 |
| RD3 | 80,604,475 | 24,181,342,500 | 150 |
| RD4 | 80,576,993 | 24,173,097,900 | 150 |
| RD5 | 80,742,706 | 24,222,811,800 | 150 |
| RD6 | 80,677,947 | 24,203,384,100 | 150 |
| RD7 | 80,579,627 | 24,173,888,100 | 150 |
| RD8 | 71,506,476 | 21,451,942,800 | 150 |
| RD9 | 78,376,031 | 23,512,809,300 | 150 |
| RL1 | 75,265,551 | 22,579,665,300 | 150 |
| RL2 | 80,533,978 | 24,160,193,400 | 150 |
| RL3 | 80,680,964 | 24,204,289,200 | 150 |
| RL4 | 80,789,803 | 24,236,940,900 | 150 |
| RL5 | 71,123,579 | 21,337,073,700 | 150 |
| RL6 | 80,792,563 | 24,237,768,900 | 150 |
| RL7 | 80,507,583 | 24,152,274,900 | 150 |
| RL8 | 68,028,790 | 20,408,637,000 | 150 |
| RL9 | 80,930,722 | 24,279,216,600 | 150 |
| RL10 | 80,829,025 | 24,248,707,500 | 150 |
| RR1 | 80,605,194 | 24,181,558,200 | 150 |
| RR2 | 69,935,733 | 20,980,719,900 | 150 |
| RR3 | 70,312,047 | 21,093,614,100 | 150 |
| RR4 | 82,287,045 | 24,686,113,500 | 150 |
| RR5 | 74,677,893 | 22,403,367,900 | 150 |
| RR6 | 67,224,248 | 20,167,274,400 | 150 |
| RR7 | 80,540,233 | 24,162,069,900 | 150 |
| RR8 | 80,618,607 | 24,185,582,100 | 150 |
| RR9 | 80,320,318 | 24,096,095,400 | 150 |
| RR10 | 80,683,284 | 24,204,985,200 | 150 |
| RR11 | 80,872,548 | 24,261,764,400 | 150 |
| RY1 | 71,592,733 | 21,477,819,900 | 150 |
| RY2 | 71,556,272 | 21,466,881,600 | 150 |
| RY3 | 72,557,049 | 21,767,114,700 | 150 |

Table S2 the countable and quantifiable traits of the three populations

| **Item** | **Value** |
| --- | --- |
| Scales | None |
| Maxillary Barbel | 2 pairs |
| Mandibular Barbel | 2 pairs |
| Dorsal Fin Rays | I, 5-6 |
| Pectoral Fin Rays | I, 11-12 |
| Pelvic Fin Rays | I, 10-12 |
| Anal Fin Rays | 34-43 |
| Body Length/Body Height | 3.6-4.7 |
| Body Length/Head Length | 3.8-4.5 |
| Body Length/Caudal Peduncle Length | 6.5-7.9 |
| Body Length/Caudal Peduncle Height | 9.7-11.6 |
| Body Length/Snout Length | 2.3-2.7 |

Table S3 Estimated migration rates and effective population sizes among three *Cranoglanis* populations

| Population Pair | Raw Migration Rate (m) | Donor Population Ne | Effective Migration Rate (Nm = m×Ne) |
| --- | --- | --- | --- |
| CH ↔ CM | 9.11E-04/  1.54E-05 | CH: 504; CM: 366 | CH→CM:~0.46; CM→CH: ~0.006; |
| CB ↔CH | 3.26E-04/  0.002039 | CB: 340; CH: 504 | CB→CH:~0.11; CH→CB: ~1.03; |
| CB ↔ CM | 1.25E-05/  1.78E-05 | CB: 340; CM: 366 | CB→CM:~0.004 CM→CB: ~0.007; |

Table S4 Descriptive statistics of individual heterozygosity indices among three *Cranoglanis* populations

| Population | Mean Heterozygosity Index | Standard Deviation (SD) | Range (Min–Max) |
| --- | --- | --- | --- |
| CH | -0.1211 | 0.0219 | -0.1949 ~ -0.0613 |
| CM | -0.0048 | 0.1352 | -0.5602 ~ 0.1115 |
| CB | 0.7072 | 0.0228 | 0.6401 ~ 0.7305 |

Table S5 the genetic diversity of the three populations were assessed by using the Cytb and COI mitochondrial molecular markers

|  | CB | CM | CH |
| --- | --- | --- | --- |
| π(Cytb) | 0.00340 | 0.00075 | 0.00330 |
| π(COⅠ) | 0.00231 | 0.00053 | 0.00181 |

Table S6 Observed and Expected Heterozygosity of Different *Cranoglanis* Populations at Variant Positions and All Positions

| Population | Variant positions | | All positions | |
| --- | --- | --- | --- | --- |
|  | **ObsHet** | **ExpHet** | **ObsHet** | **ExpHet** |
| RD | 0.2988 | 0.2766 | 1.3268E-04 | 1.2283E-04 |
| HN | 0.2647 | 0.2332 | 1.1754E-04 | 1.0355E-04 |
| RL | 0.2963 | 0.2816 | 1.3157E-04 | 1.2505E-04 |
| RR | 0.2979 | 0.283 | 1.3228E-04 | 1.2567E-04 |
| RY | 0.2883 | 0.2435 | 1.2802E-04 | 1.0813E-04 |
| ZH | 0.078 | 0.064 | 3.4636E-05 | 2.8419E-05 |
| ZL | 0.0768 | 0.0687 | 3.4103E-05 | 3.0507E-05 |


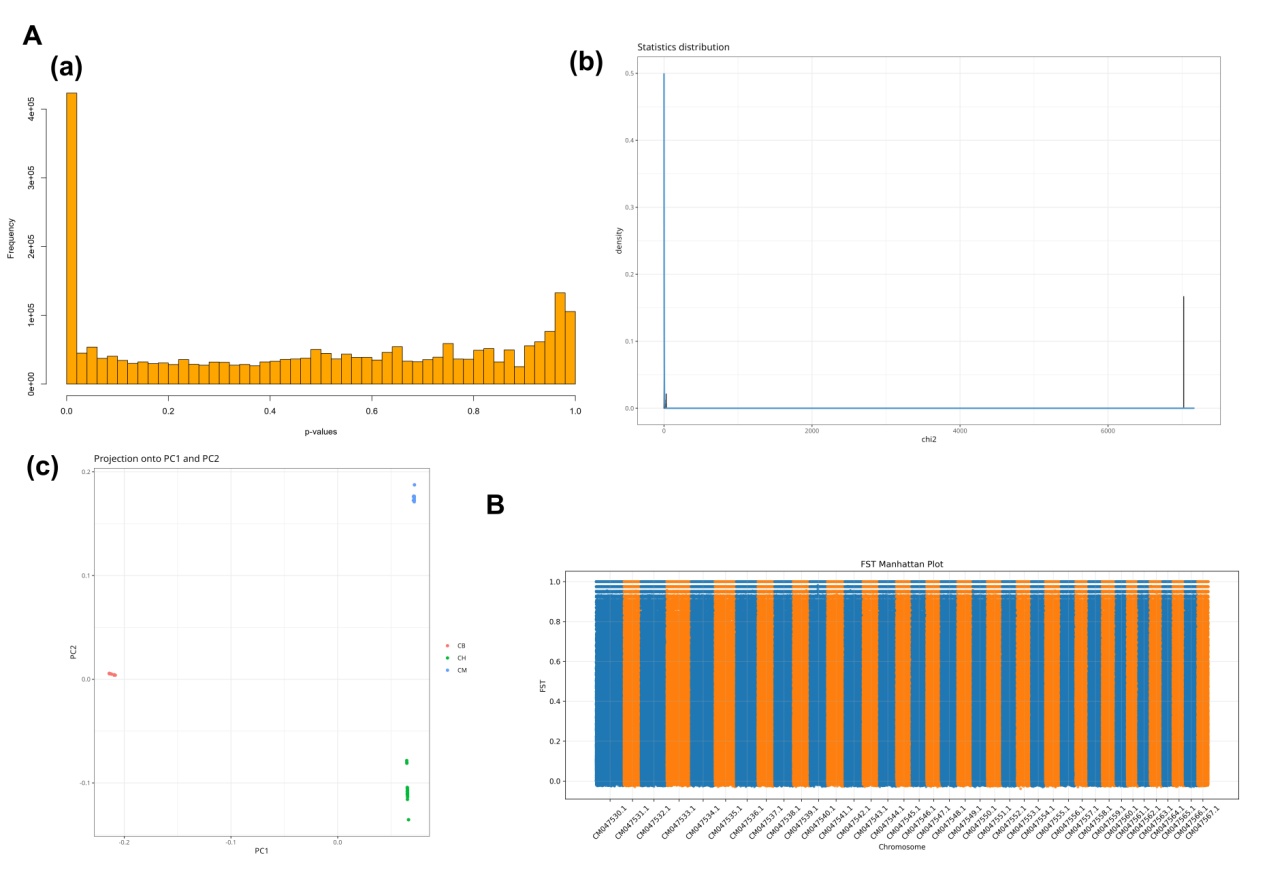


Figure S1 Genetic analyses of population divergence and adaptive loci. (A) pcadapt results for detecting adaptive genetic variation:(a) p-value frequency histogram of pcadapt output. (b) Distribution of chi-squared (χ²) statistics from pcadapt. (c) Projection of samples onto the first two principal components (PC1 and PC2) from pcadapt. (B) *F_ST_* Manhattan plot of filtered single nucleotide polymorphisms (SNPs).


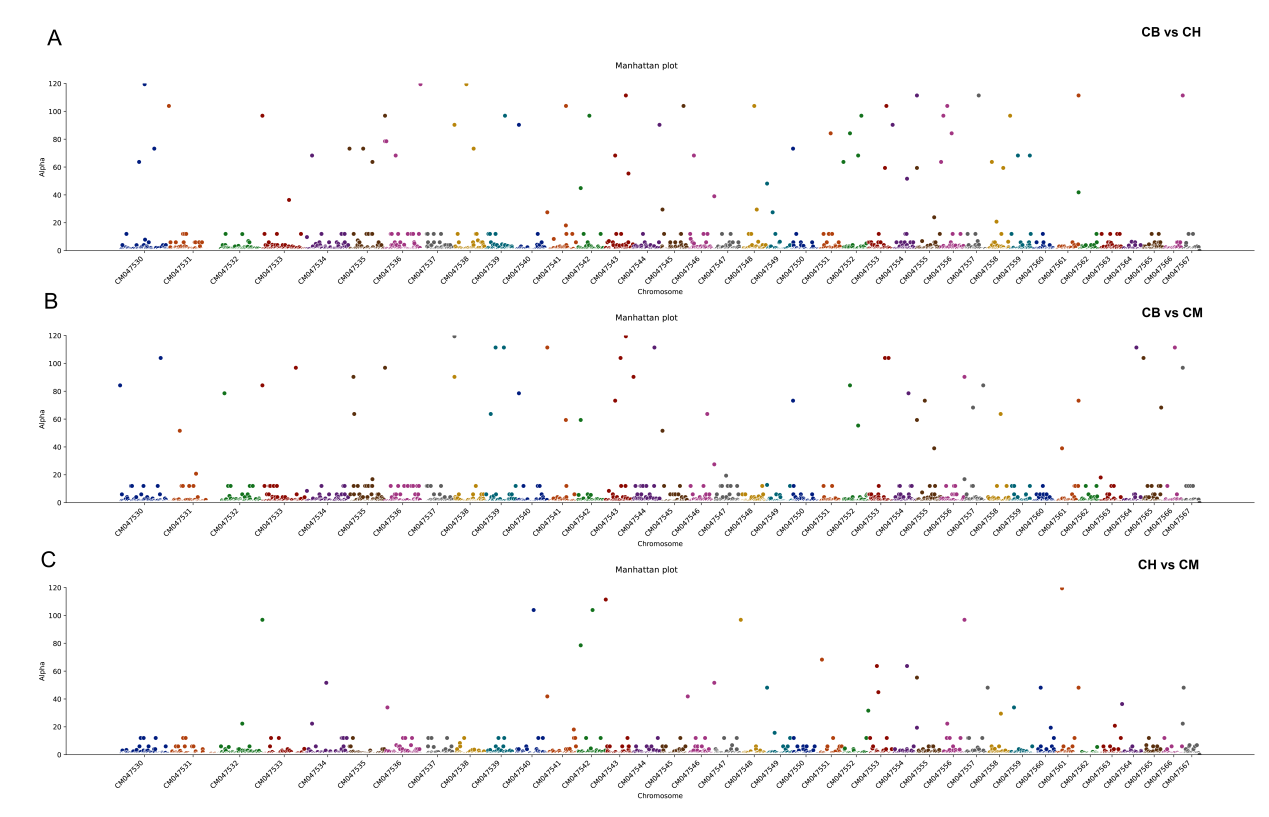


Figure S2 Window-based *F_ST_* Manhattan plots of three population pairs in *Cranoglanis*. (A), CB vs. CH; (B), CB vs CM; (C), CH vs CM.


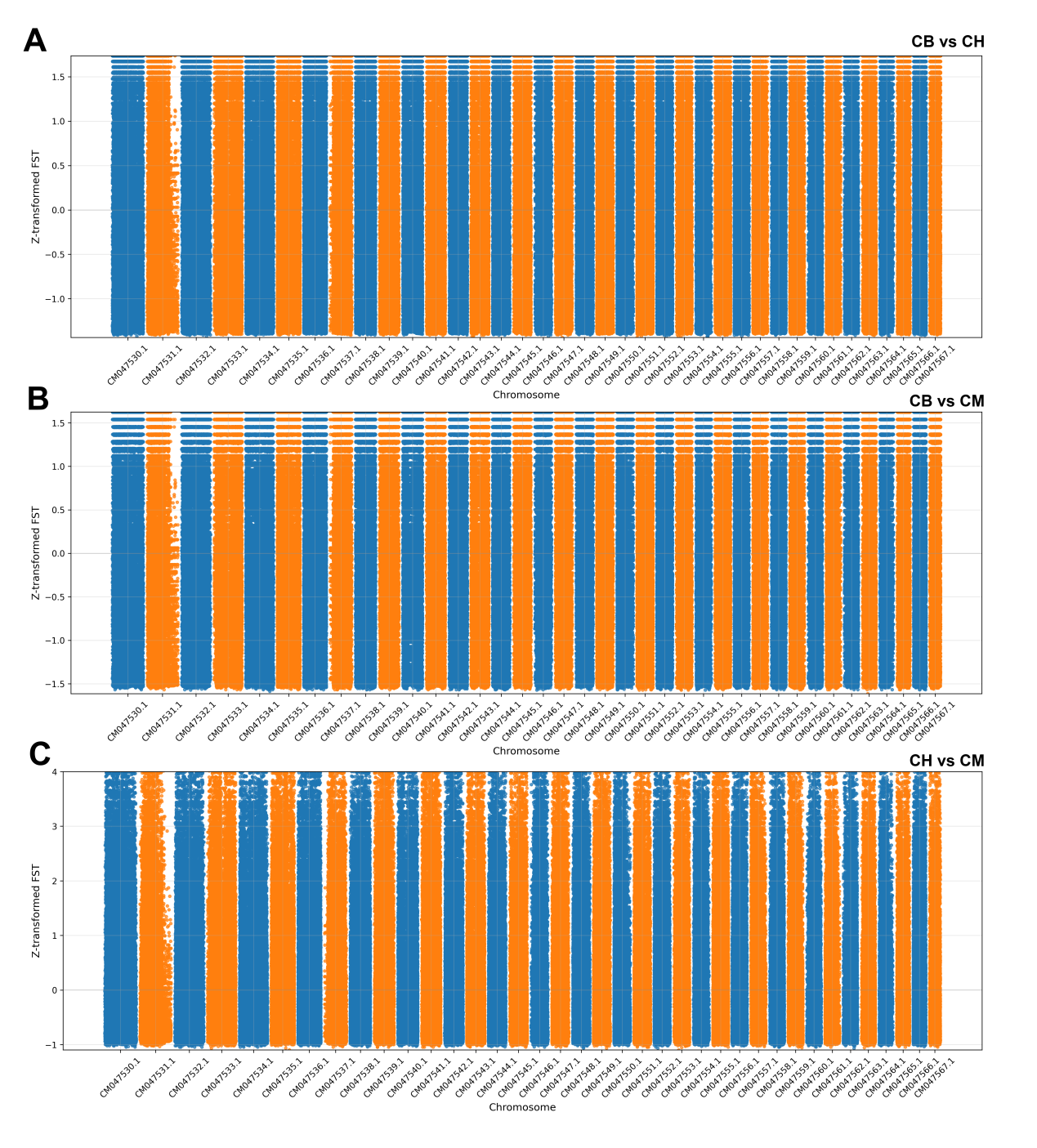


Figure S3 Z-transformed *F_ST_* (Z-*F_ST_*) Manhattan plots displaying genome-wide differentiation between three *Cranoglanis* population pairs. Chromosomes are colored alternately blue and orange for visual clarity.

(A), CB vs. CH; (B), CB vs CM; (C), CH vs CM.


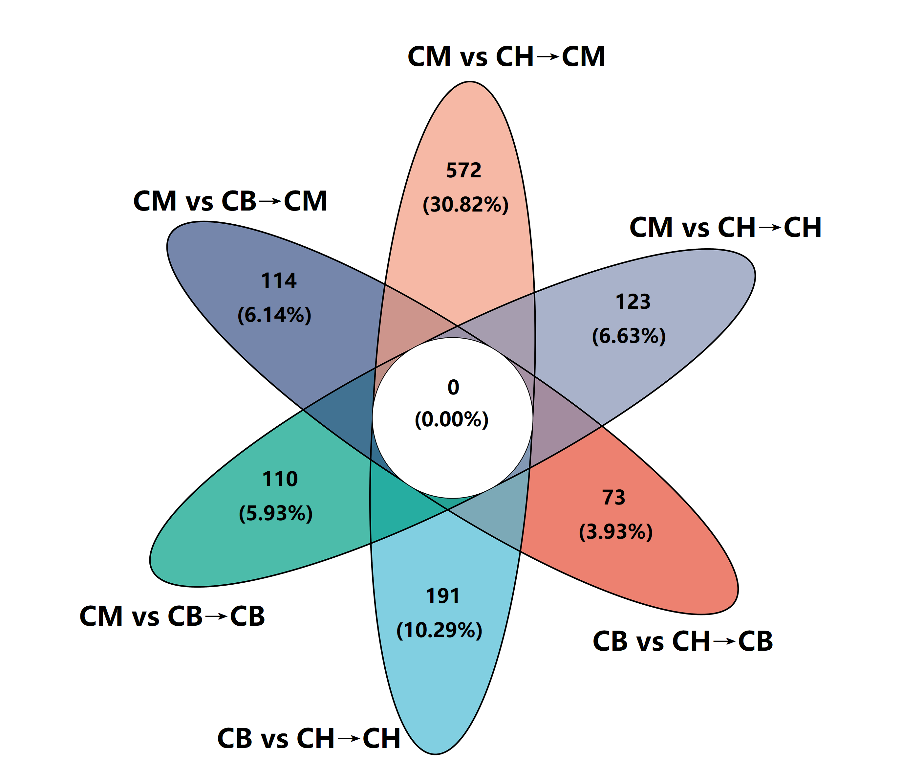


Figure S4 Venn diagram of selected genes numbers.


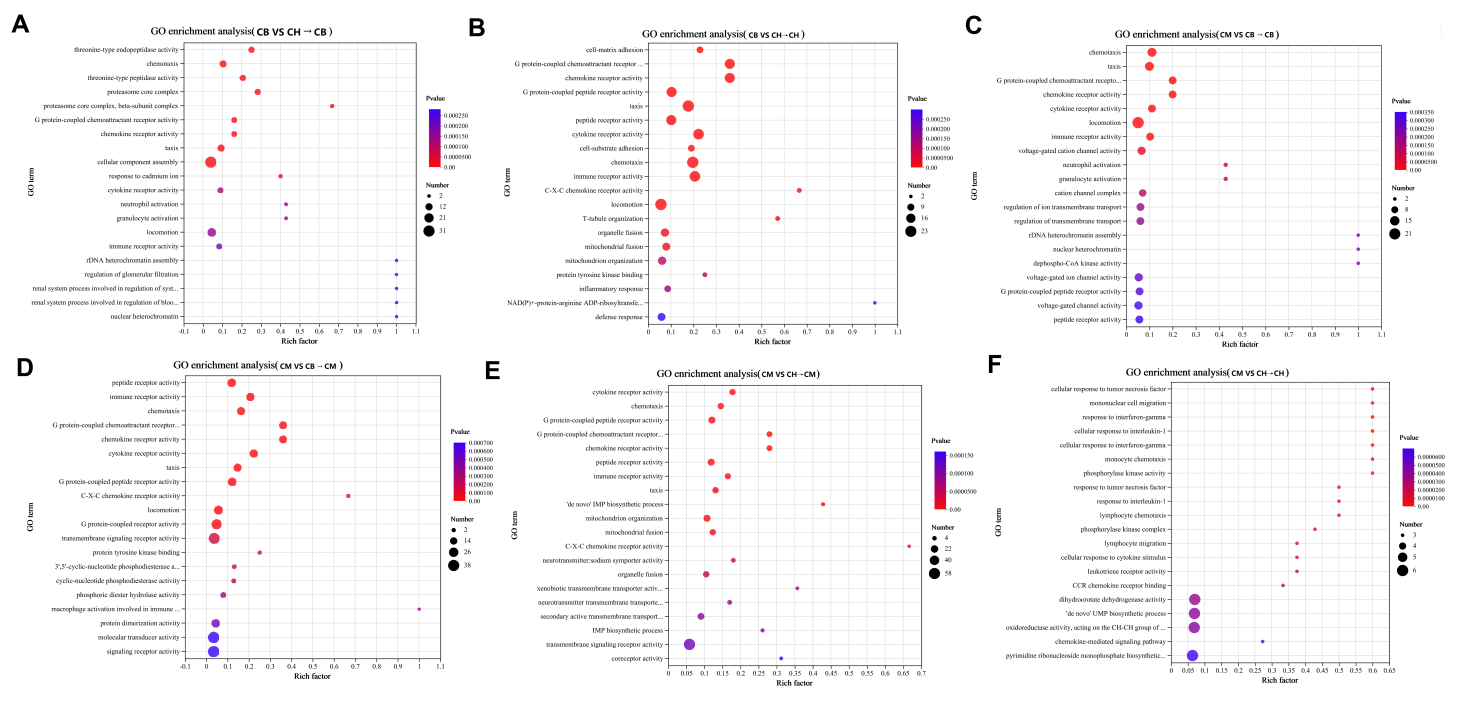


Figure S5 Enrichment analysis of GO terms of differentially selected genes among three *Cranoglanis* populations. A and B show enriched terms for selected genes in CB vs CH. C and D display enriched terms for CB vs CM. E and F present enriched terms for CM and CH.
