# Supplementary material for: Whole-genome resequencing reveals genetic divergence, local adaptation, and conservation priorities in three helmet catfishes (complex Cranoglanis bouderius)
Source: BMC Genomics. 2026 Apr 14;27:500. doi: 10.1186/s12864-026-12843-3 (PMC13188337; doi:10.1186/s12864-026-12843-3)
Supplement: Supplementary file 3 — Supplementary Material 3. [file 12864_2026_12843_MOESM3_ESM.docx]

| INDV | O(HOM) | E(HOM) | N_SITES | F |
| --- | --- | --- | --- | --- |
| D1A | 2075269 | 1817972.2 | 2500402 | 0.37703 |
| D2A | 2074895 | 1817972.2 | 2500402 | 0.37648 |
| D3A | 2085775 | 1817972.2 | 2500402 | 0.39243 |
| D4A | 2087244 | 1817972.2 | 2500402 | 0.39458 |
| D5A | 2082838 | 1817972.2 | 2500402 | 0.38812 |
| D6A | 2084783 | 1817972.2 | 2500402 | 0.39097 |
| D7A | 2080437 | 1817972.2 | 2500402 | 0.38460 |
| D8A | 2071271 | 1817972.2 | 2500402 | 0.37117 |
| D9A | 2077994 | 1817972.2 | 2500402 | 0.38102 |
| H1 | 2135756 | 1817972.2 | 2500402 | 0.46567 |
| H10 | 2089069 | 1817972.2 | 2500402 | 0.39725 |
| H11 | 2093725 | 1817972.2 | 2500402 | 0.40407 |
| H12 | 1877918 | 1817972.2 | 2500402 | 0.08784 |
| H13 | 2119469 | 1817972.2 | 2500402 | 0.44180 |
| H14 | 2055603 | 1817972.2 | 2500402 | 0.34821 |
| H15 | 2133354 | 1817972.2 | 2500402 | 0.46215 |
| H16 | 2114200 | 1817972.2 | 2500402 | 0.43408 |
| H17 | 2132431 | 1817972.2 | 2500402 | 0.46079 |
| H18 | 2067189 | 1817972.2 | 2500402 | 0.36519 |
| H19 | 2142596 | 1817972.2 | 2500402 | 0.47569 |
| H2 | 2130025 | 1817972.2 | 2500402 | 0.45727 |
| H20 | 2082078 | 1817972.2 | 2500402 | 0.38701 |
| H3 | 2096235 | 1817972.2 | 2500402 | 0.40775 |
| H4 | 2137112 | 1817972.2 | 2500402 | 0.46765 |
| H5 | 2101745 | 1817972.2 | 2500402 | 0.41583 |
| H6 | 2139039 | 1817972.2 | 2500402 | 0.47048 |
| H7 | 2106480 | 1817972.2 | 2500402 | 0.42277 |
| H8 | 2150217 | 1817972.2 | 2500402 | 0.48686 |
| H9 | 2124411 | 1817972.2 | 2500402 | 0.44904 |
| L10A | 2084722 | 1817972.2 | 2500402 | 0.39088 |
| L1A | 2085653 | 1817972.2 | 2500402 | 0.39225 |
| L2A | 2099238 | 1817972.2 | 2500402 | 0.41215 |
| L3A | 2076117 | 1817972.2 | 2500402 | 0.37827 |
| L4A | 2087404 | 1817972.2 | 2500402 | 0.39481 |
| L5A | 2085830 | 1817972.2 | 2500402 | 0.39251 |
| L6A | 2071905 | 1817972.2 | 2500402 | 0.37210 |
| L7A | 2083479 | 1817972.2 | 2500402 | 0.38906 |
| L8A | 2083914 | 1817972.2 | 2500402 | 0.38970 |
| L9A | 2084637 | 1817972.2 | 2500402 | 0.39076 |
| R10A | 2085636 | 1817972.2 | 2500402 | 0.39222 |
| R11A | 2089252 | 1817972.2 | 2500402 | 0.39752 |
| R12A | 2087640 | 1817972.2 | 2500402 | 0.39516 |
| R1A | 2095272 | 1817972.2 | 2500402 | 0.40634 |
| R2A | 2079128 | 1817972.2 | 2500402 | 0.38269 |
| R3A | 2056183 | 1817972.2 | 2500402 | 0.34906 |
| R4A | 2082169 | 1817972.2 | 2500402 | 0.38714 |
| R5A | 2069182 | 1817972.2 | 2500402 | 0.36811 |
| R7A | 2084399 | 1817972.2 | 2500402 | 0.39041 |
| R8A | 2080589 | 1817972.2 | 2500402 | 0.38483 |
| R9A | 2088583 | 1817972.2 | 2500402 | 0.39654 |
| RYH1 | 2088640 | 1817972.2 | 2500402 | 0.39662 |
| RYH2 | 2092936 | 1817972.2 | 2500402 | 0.40292 |
| RYH3 | 2106005 | 1817972.2 | 2500402 | 0.42207 |
| ZH1 | 2082874 | 1817972.2 | 2500402 | 0.38817 |
| ZH10 | 2012741 | 1817972.2 | 2500402 | 0.28540 |
| ZH2 | 2080693 | 1817972.2 | 2500402 | 0.38498 |
| ZH5 | 2041282 | 1817972.2 | 2500402 | 0.32723 |
| ZH6 | 2060072 | 1817972.2 | 2500402 | 0.35476 |
| ZH8 | 1931688 | 1817972.2 | 2500402 | 0.16663 |
| ZH9 | 2068293 | 1817972.2 | 2500402 | 0.36681 |
| ZL1 | 2081564 | 1817972.2 | 2500402 | 0.38625 |
| ZL10 | 2082796 | 1817972.2 | 2500402 | 0.38806 |
| ZL2 | 2067508 | 1817972.2 | 2500402 | 0.36566 |
| ZL3 | 2064795 | 1817972.2 | 2500402 | 0.36168 |
| ZL4 | 2089682 | 1817972.2 | 2500402 | 0.39815 |
| ZL5 | 2004126 | 1817972.2 | 2500402 | 0.27278 |
| ZL6 | 2087376 | 1817972.2 | 2500402 | 0.39477 |
| ZL7 | 2056763 | 1817972.2 | 2500402 | 0.34991 |
|  |  |  |  |  |
| ZL8 | 2063034 | 1817972.2 | 2500402 | 0.35910 |
|  |  |  |  |  |
|  |  |  |  |  |
|  |  |  |  |  |
|  |  |  |  |  |
|  |  |  |  |  |
|  |  |  |  |  |
|  |  |  |  |  |
|  |  |  |  |  |
|  |  |  |  |  |
|  |  |  |  |  |
|  |  |  |  |  |
|  |  |  |  |  |
|  |  |  |  |  |
|  |  |  |  |  |
|  |  |  |  |  |
|  |  |  |  |  |
|  |  |  |  |  |
|  |  |  |  |  |
|  |  |  |  |  |
|  |  |  |  |  |
|  |  |  |  |  |
|  |  |  |  |  |
|  |  |  |  |  |
|  |  |  |  |  |
|  |  |  |  |  |
|  |  |  |  |  |
|  |  |  |  |  |
|  |  |  |  |  |
|  |  |  |  |  |
|  |  |  |  |  |
|  |  |  |  |  |
|  |  |  |  |  |
|  |  |  |  |  |
|  |  |  |  |  |
|  |  |  |  |  |
|  |  |  |  |  |
|  |  |  |  |  |
|  |  |  |  |  |
|  |  |  |  |  |
|  |  |  |  |  |
|  |  |  |  |  |
|  |  |  |  |  |
|  |  |  |  |  |
|  |  |  |  |  |
|  |  |  |  |  |
|  |  |  |  |  |
|  |  |  |  |  |
|  |  |  |  |  |
|  |  |  |  |  |
|  |  |  |  |  |
|  |  |  |  |  |
|  |  |  |  |  |
|  |  |  |  |  |
|  |  |  |  |  |
|  |  |  |  |  |
|  |  |  |  |  |
|  |  |  |  |  |
|  |  |  |  |  |
|  |  |  |  |  |
|  |  |  |  |  |
|  |  |  |  |  |
|  |  |  |  |  |
|  |  |  |  |  |
|  |  |  |  |  |
|  |  |  |  |  |
|  |  |  |  |  |
|  |  |  |  |  |
|  |  |  |  |  |
|  |  |  |  |  |
|  |  |  |  |  |
|  |  |  |  |  |
|  |  |  |  |  |
|  |  |  |  |  |
| ZL9 | 2069622 | 1817972.2 | 2500402 | 0.36876 |
